# Beta-lactamase dependent and independent evolutionary paths to high-level ampicillin resistance

**DOI:** 10.1038/s41467-024-49621-2

**Published:** 2024-06-25

**Authors:** Rotem Gross, Idan Yelin, Viktória Lázár, Manoshi Sen Datta, Roy Kishony

**Affiliations:** 1 Faculty of Biology, Technion–Israel Institute of Technology, Haifa, Israel; 2 Faculty of Computer Science, Technion–Israel Institute of Technology, Haifa, Israel; 3 Faculty of Biomedical Engineering, Technion–Israel Institute of Technology, Haifa, Israel; 4 Present Address: HCEMM-BRC Pharmacodynamic Drug Interaction Research Group, Szeged, Hungary; 5 Present Address: Synthetic and Systems Biology Unit, Institute of Biochemistry, HUN-REN Biological Research Centre, Szeged, Hungary; 6 Present Address: The California Institute for Quantitative Biosciences, University of California, Berkeley, Berkeley, CA USA

**Keywords:** Experimental evolution, Bacterial genomics, Bacterial evolution, Antimicrobial resistance

## Abstract

The incidence of beta-lactam resistance among clinical isolates is a major health concern. A key method to study the emergence of antibiotic resistance is adaptive laboratory evolution. However, in the case of the beta-lactam ampicillin, bacteria evolved in laboratory settings do not recapitulate clinical-like resistance levels, hindering efforts to identify major evolutionary paths and their dependency on genetic background. Here, we used the Microbial Evolution and Growth Arena (MEGA) plate to select ampicillin-resistant Escherichia coli mutants with varying degrees of resistance. Whole-genome sequencing of resistant isolates revealed that ampicillin resistance was acquired via a combination of single-point mutations and amplification of the gene encoding beta-lactamase AmpC. However, blocking AmpC-mediated resistance revealed latent adaptive pathways: strains deleted for ampC were able to adapt through combinations of changes in genes involved in multidrug resistance encoding efflux pumps, transcriptional regulators, and porins. Our results reveal that combinations of distinct genetic mutations, accessible at large population sizes, can drive high-level resistance to ampicillin even independently of beta-lactamases.

## Abstract (web summary)

Bacterial resistance to beta-lactam antibiotics is on the rise, but laboratory evolution studies do not always recapitulate clinical resistance levels. Here, the authors select Escherichia coli mutants with varying degrees of beta-lactam resistance, showing that combinations of distinct genetic mutations, accessible at large population sizes, can drive high-level resistance independently of beta-lactamases.

## Introduction

Beta-lactams are utilised to treat a wide variety of infections, but their clinical effectiveness is often jeopardised by beta-lactam-resistant strains^1–5^. These cell wall inhibitors are the most widely prescribed antibiotic class^6,7^, accounting for almost 65% of global antibiotic use in the clinic^7–10^. Of particular concern is ampicillin, an extended-spectrum beta-lactam used against various medically important pathogens. In recent years, these pathogens have evolved high levels of resistance^11–17^, well above their resistance-defining antibiotic concentration^18,19^.

The evolutionary pathways available for bacteria adapting to ampicillin are of great interest and have therefore been extensively studied^20–26^. Laboratory evolution experiments coupled with whole-genome sequencing revealed distinct genetic alterations leading to ampicillin resistance^20,23,24^. The overexpression of beta-lactamases is one of the most widespread mechanisms of resistance to beta-lactams^27,28^. In particular, the AmpC beta-lactamase is a clinically important ­cephalosporinase encoded on the chromosomes of many of the *Enterobacteriaceae*, mediating resistance to a wide range of beta-lactams including ampicillin^29,30^. In *E. coli*, *ampC* is typically silent or expressed at very low basal levels^27,31,32^, but its expression is often increased in laboratory-evolved strains^32–34^ due to promoter mutations or gene amplification^30,35,36^. Mutations in other genes, including porin-coding genes such as *ompF*, *ompC*, ampicillin targets *ftsI, mrdA*, and efflux pumps *acrAB, marR*, can also contribute to resistance in laboratory experiments^20,23,24^, and can even lead to intermediate resistance on their own when challenged by beta-lactams not hydrolyzable by ampC^37,38^.

Notably, in these laboratory evolution experiments, in contrast to the clinic, bacteria adapting to ampicillin usually only reach low to intermediate resistance levels^20–26^. Therefore, less is known about mutational combinations and trajectories leading to high-level ampicillin resistance (>50-fold increase in resistance compared to the ancestor), and even less is known about how such evolutionary trajectories may depend on the presence of a beta-lactamase gene.

The microbial evolution and growth arena (MEGA-) plate is a tool designed to challenge large bacterial populations with antibiotic drugs and directly observe their evolution as they gradually adapt to escalating antibiotic concentrations. It has been previously used to study the multi-step adaptive evolution towards high level resistance to trimethoprim and ciprofloxacin^39^. We hypothesised that due to the gradual increase in drug concentration and large population size, this experimental setting will allow the evolution of ampicillin resistance at clinically observed levels through diverse paths, possibly recapitulating those observed clinically. Evolving wild type and *ampC*-deleted ancestral strains and sampling isolates along the adaptive paths can further reveal adaptive pathways in the presence and absence of the dedicated beta-lactamase gene.

## Results

### Adaptive laboratory evolution in a MEGA-plate

**Fig. 1:**
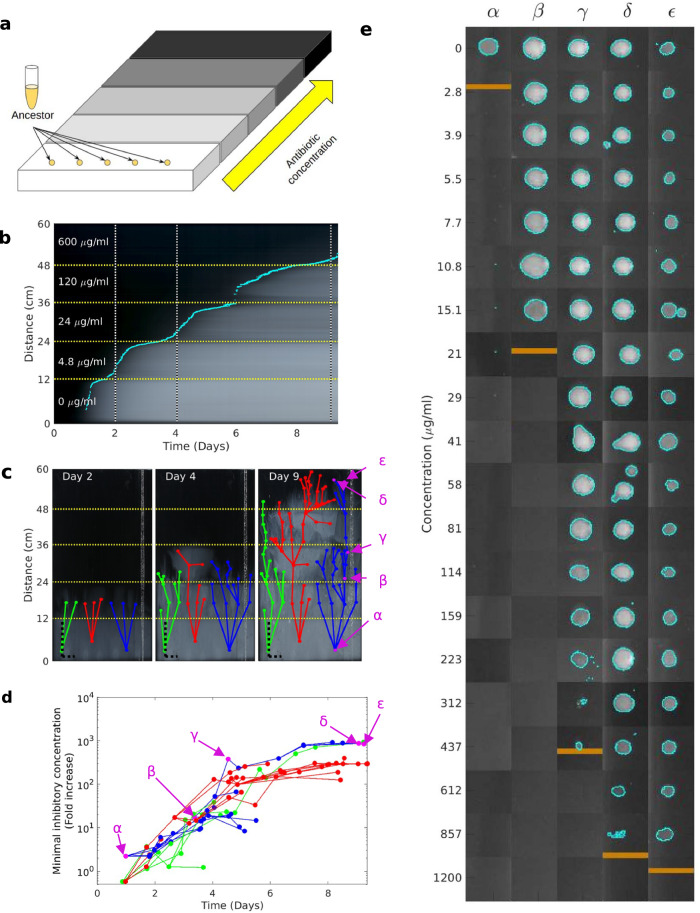
MEGA-plate experimental setting allows the evolution of high-level ampicillin resistance. **a** Schematic representation of the MEGA-plate setting, where bacteria swim against a stepwise gradient of increasing ampicillin concentrations. **b** A kymograph of MEGA-plate experiment A, showing for each time-point, for each distance from the starting line, the 90th percentile of pixel intensity. Cyan dots represent the farthest point reached by bacteria at each time point as inferred from image intensity. Vertical black-and-white dotted lines indicate three time points (days 2, 4, and 9) corresponding to still images presented in (**c**). **c** Still images of the MEGA-plate at three time points (days 2, 4, and 9), indicating the locations of isolates sampled from three distinct lineages (red, green, and blue connected points). For presentation purposes, images are compressed along the horizontal axis, as indicated by the horizontal and vertical dashed black scale, both representing 10 cm. **d** The increase in resistance levels as a function of time for isolates from three distinct lineages (green, red, and blue) evolving on the MEGA-plate. In both (**b**, **c**), concentration steps are indicated by yellow dashed lines. **e** MIC measurements for the 5 isolates indicated in panels c and d (labelled ɑ, β, γ, ε, δ, corresponding to isolates 3, 21, 47, 89, 90 in Supplementary Fig. 1a); cyan contour indicates detected colonies on the antibiotic gradient, MIC is indicated by horizontal orange lines. Source data are provided as a Source Data file.

We started by challenging *E. coli* in a 5-step MEGA-plate. A wild type ancestral *E. coli* strain was inoculated onto a MEGA-plate patterned with 5 increasing concentrations of ampicillin, starting with 4.8 µg/ml (twice the ancestral Minimal Inhibitory Concentration, MIC) and rising 5-fold at each step (Fig. 1a, c, d; Methods, Exp. A; Supplementary Table 1; see movie 1 available at 10.5281/zenodo.7950385). Time-lapse imaging revealed distinct lineages propagating through the antibiotic gradient ultimately reaching, within 9 days, the area of highest concentration, 600 µg/ml (Fig. 1c, d). Next, we collected isolates from various locations on the MEGA-plate and measured their MIC by plating on a set of agar plates, containing different ampicillin concentrations, and quantifying growth by automated image analysis (Methods; Fig. 1e; 279 isolates, 3 colonies from 93 sampled locations; Supplementary Fig. 1). These MIC measurements revealed the adaptive trajectories of resistance as a function of time and indicated that resistance evolved to levels similar to those observed in resistant clinical isolates^20,40^, exceeding 1000 µg/ml, a more than 400-fold increase compared to the ancestor (Fig. 1b; MIC was measured on solid, Methods). Similar resistance levels were observed in another similar repeat MEGA-plate experiment (Exp. B, up to 5000 µg/ml; Supplementary Fig. 2; Supplementary Table 1; see movie 2 available at 10.5281/zenodo.7950385; 94 isolates). Thus, in contrast to prior experiments, the MEGA-plate laboratory-acquired resistance levels recapitulate the resistance level of clinical strains^18,19^.

### The impact of beta-lactamase on adaptive potential and pathways

**Fig. 2:**
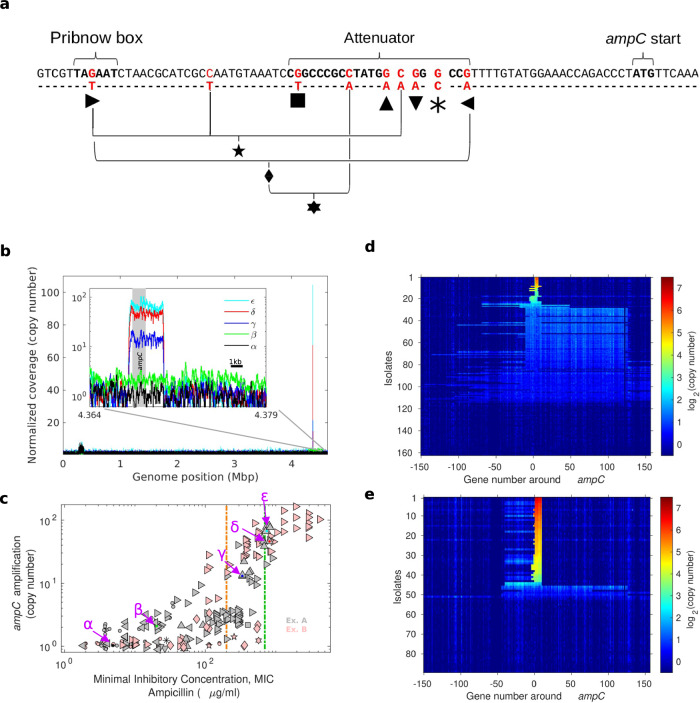
High-level ampicillin resistance is achieved by increased expression of AmpC through promoter mutations and increasingly focusing gene amplifications. **a** Promoter sequence of *ampC* gene, indicating all mutated loci observed in isolates from Experiments A and B (red letters). Each observed change, of either a single or multiple nucleotides, is indicated by a unique symbol. For combinations, a symbol indicates a change in all loci connected to it by black lines, e.g., the hexagram symbol indicates a concomitant change in three loci: two G > A transitions marked by sideways-pointing triangles and a single C > A transversion. **b** The normalised nucleotide copy number across the genome of the same isolates appearing in Fig. 1. Inset shows a zoom-in on the region around the *ampC* gene (grey box). Black scale bar represents 1000 bp. **c** The MIC and the copy number of the *ampC* locus are shown for each isolate (Exp A, *n* = 148, Grey; Exp B, *n* = 89; Pink). Shapes indicate the promoter mutation (as defined in **a**). Circle represents no *ampC*-associated mutation. Vertical lines denote antibiotic concentrations beyond which the *ampC* gene is always either amplified or carries a promoter mutation (orange) or is highly amplified in all isolates (green). **d**, **e** Normalised gene copy number of the region surrounding the *ampC* locus (150 genes from each side). Isolates ordered by the copy number of the *ampC* locus itself, for MEGA-plate Experiments A (**d**) and B (**e**). Source data are provided as a Source Data file.

Whole-genome sequencing revealed that in all high-resistance isolates, the *ampC* locus had either promoter mutations or genomic amplifications, or both. To uncover the adaptive genetic changes underlying increased ampicillin resistance, we whole-genome sequenced 240 isolates from Experiments A and B above (Methods). Across all isolates, we identified 45 single-nucleotide polymorphisms (SNPs) in 34 different genes, including 5 SNPs at the attenuator site and Pribnow box upstream of the *ampC* gene, known to increase expression (Supplementary Dataset 5; Fig. 2a)^41,42^. Additionally, we identified multiple independently arising amplifications of the *ampC* locus (Fig. 2b–e). High-level *ampC* gene amplification (>20 copies) was observed in all isolates with MIC above 700 µg/ml (green line, Fig. 2c). These amplifications were increasingly focused on the resistance conferring gene; higher copy number of the *ampC* gene was associated with a narrower amplified region around the *ampC* locus (Fig. 2d, e). In total, all isolates that reached ampicillin resistance above 200 µg/ml (orange line, Fig. 2c) carried either a mutation in the promoter region of *ampC* or amplification of the *ampC* gene, highlighting the importance of *ampC* for high-level ampicillin resistance.

**Fig. 3:**
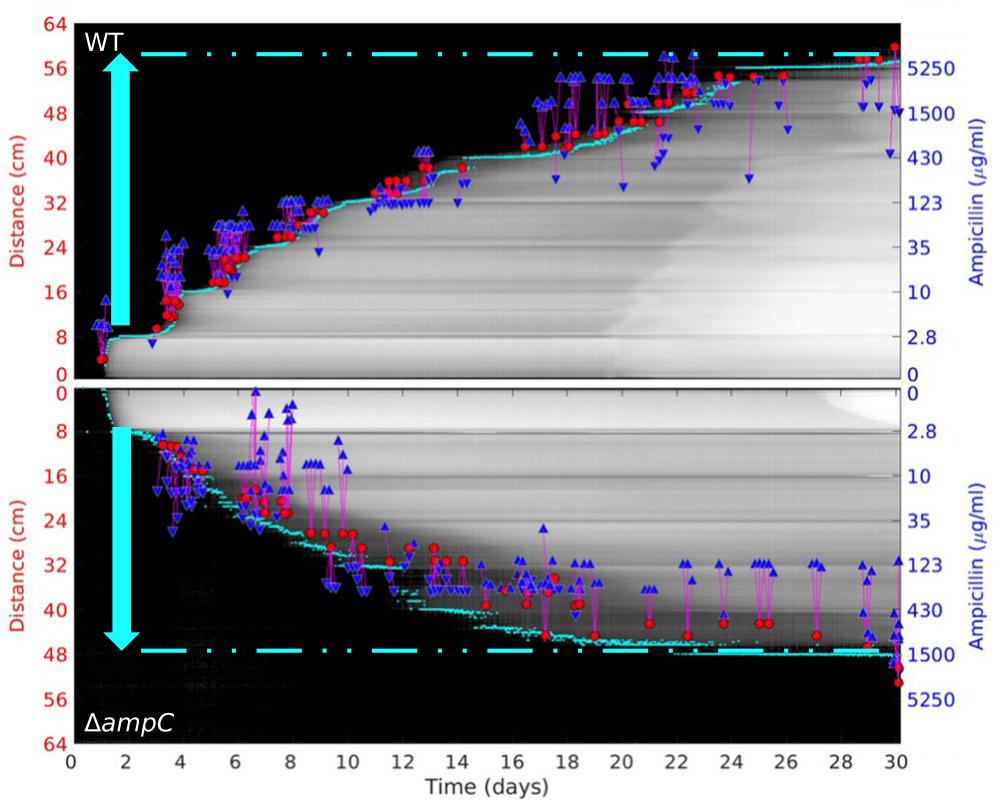
*ampC* deleted strain reached slightly reduced, but still high, ampicillin resistance. Kymographs showing the propagation of the evolving bacterial front of the wild type strain (top; Exp. C) and the *ampC*-deleted strain (bottom; Exp. D) challenged by the same ampicillin gradient (blue, right). Cyan dots represent the inferred front at each time point. Cyan arrow and dashed line indicate the delta between the first selective barrier and the farthest distance reached by the advancing front. Collected isolates are represented by red dots indicating their distance from the inoculation point (left y-axis) and their appearance time (x-axis). Appearance time is determined for each 9 × 9 pixel region as it exceeds an intensity threshold; see Methods. Blue triangles represent the MIC measurements of a given isolate (typically 3 isolates per each sampled location). For each isolate, a magenta vertical line highlights the difference between the measured MIC and the ampicillin concentration expected at the given region from where the samples were isolated. Source data are provided as a Source Data file.

Despite the observed association of high-level ampicillin resistance with *ampC* promoter mutations or amplifications, we find that high-level resistance was attainable even in absence of *ampC*. To reveal the adaptive potential of *E. coli* to ampicillin resistance in the absence of the *ampC* beta-lactamase, we designed a MEGA-plate experiment in which we evolved in parallel both the wild type and an *ampC*-deleted ancestral strains on a single MEGA-plate device divided into equal-width lanes with identical ampicillin steps (Exps. C and D; 9 steps of 3.5-fold increase starting from 10 µg/ml; Methods; Supplementary Table 1; see movies 3 and 4 available at 10.5281/zenodo.7950385). Tracking the adaptive front of these evolving strains, we found that within about 15 days, both strains reached the sixth step, in which ampicillin concentrations were as high as 1500 µg/ml (Fig. 3). The evolved *ampC*-deleted strain plateaued at this already high level, yet the wild type strain kept evolving, ultimately reaching the seventh step of 3.5 times the ampicillin concentration (5250 µg/ml). A similar trend was also observed when considering the level of resistance of isolates sampled from each of these evolving populations. The measured MICs were as high as 2048 µg/ml for evolved isolates of the *ampC*-deleted strain and exceeded 8192 µg/ml for evolved isolates of the wild type, indicating that the adaptive potential of the *ampC* deleted strain was only slightly reduced compared with the adaptive potential of the wild type^19,43^. We noticed that while the measured resistance for the wildtype strain was often higher than the ampicillin concentration expected according to the position on the MEGA-plate from which they were sampled, this was not the case for the *ampC*-deleted strain. We hypothesised that this reflects differences in the type of mutations underlying resistance in each of these strains, and possibly related to the higher evolvability of *ampC* gene amplifications.

### Combinations of mutations drive ampicillin resistance in the absence of *ampC* beta-lactamase

**Fig. 4:**
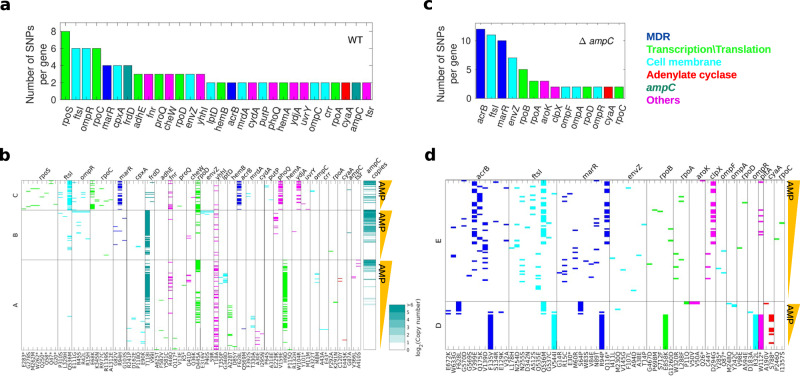
Strains deleted for *ampC* evolved high-level ampicillin resistance through combinations of multiple mutations in genes involved in multidrug resistance. The number of mutated loci in each gene for (**a**) the wild type strain and (**c**) the *ampC*-deleted strain. Genes were included in the analysis if at least two different SNPs were observed for them, or if the same SNP occurred independently in more than one evolution experiment. Genes are grouped by function (see legend). **b**, **d** SNPs (columns) observed in each isolate (rows). In (**b**), *ampC* copy number is indicated in heatmap. Isolates are grouped by experiment and sorted by their measured ampicillin MIC. Source data are provided as a Source Data file.

Whole-genome sequencing of the evolved wild type and *ampC* deleted strains allowed identifying genetic pathways to high-level resistance. We identified SNPs among wild type evolved isolates (Exps. A, B, C; a total of 304 isolates) and among *ampC* deleted isolates (Exp. D above, as well as a repeat experiment, Exp. E, Supplementary Fig. 3; Supplementary Table 1; see movie 5 available at 10.5281/zenodo.7950385; Methods; a total 116 isolates). In total, considering mutations within open reading frames only, the set of wild type descendants had accumulated 136 SNPs within a set of 85 mutated genes and the descendants of the *ampC* deleted strain had 100 SNPs spread across 50 genes (Supplementary Datasets 5, 6). Taking parallel evolution as a signal for selection^44,45^, we identified genes mutated more than expected by chance, suggesting positive selection (genes with 2 or more SNPs; Supplementary Fig. 4). To ask whether mutations in specific genes might limit further evolution (dead-end mutations), we analysed whether mutations in this gene were enriched for being identified only in isolates with the same resistance level (as would be expected for dead-end mutations; Methods; Supplementary Fig. 5). This analysis revealed several genes, most prominently *envZ*, whose mutations appeared only at specific resistance level, not leading to further evolution (Supplementary Fig. 5; *p* = 0.004 for *envZ*, marginally significant when correcting for multiple hypotheses, 37 genes). Clustering genes by function, we found that both the wild type and *ampC* deleted strains shared similar adaptive pathways, consisting of mutations in *acrB* and *marR (*multi-drug resistance genes*)* in *ftsI, envZ, ompC, ompR* (membrane-related genes), in *rpoA, rpoC*, and *rpoD* (RNA polymerase related genes) and in *cyaA* (adenylate cyclase related gene; Fig. 4a–d). These results thereby suggest that even in the absence of the *ampC* gene, bacteria can still become resistant through adaptive pathways involving the accumulation of multiple adaptive mutations.

### Large bacterial population size is critical for adaptation to ampicillin

Laboratory-evolved resistance is dependent on large bacterial population size. Given the specificity of mutations identified as important for resistance, we hypothesised that evolution in previous laboratory experiments may have been constrained by effective population size and mutation availability and that the high-level resistance reached in the MEGA-plate setting can be at least partially explained by the large population size allowed in the device. To test this hypothesis, we performed a serial passage evolution experiment in three different volumes (120 µl, 600 µl, and 8 ml), starting from the same ancestral wild type *E. coli*. For the two evolution experiments performed in the low and intermediate volumes (120 µl, and 600 µl), we observed a rapid stagnation of the evolved lines at an intermediate ampicillin resistance level, consistent with the results of previous studies (Supplementary Fig. 6)^20,24^. The resistance level of these evolved lines plateaued after 6 days at (30.48 ± 13.08 µg/ml, low; 33.06 ± 16.95 µg/ml, medium) an increase of only 10-fold compared to the ancestor. In contrast, during the serial passage experiment performed in high volume (8 ml), bacteria continuously increased their resistance level for at least 8 days and only then plateaued at a much higher ampicillin concentration, 753.9 ± 242.8 µg/ml, about 250 fold increase compared to the ancestor strain suggesting that evolution in small volumes is mutation limited (Supplementary Fig. 7a). Yet, even in this higher volume, resistance was lower than observed in the MEGA-plate, likely reflecting an even higher effective population size of the MEGA-plate and possibly more gradual and genetically diverse adaptive dynamics (Supplementary Fig. 7b).

## Discussion

To conclude, challenging a large bacterial population with increasing concentrations of ampicillin in a MEGA-plate setting, we tracked the adaptation of multiple diverging *E. coli* lineages, reaching high, clinical-like, resistance, beyond what was previously observed in laboratory evolution. This evolution of high-level resistance required large population sizes; in well-mixed experiments, populations evolving in small volumes stagnated at an intermediate level of resistance, suggesting that multiple rare mutations are required for adaptation. Indeed, we found that isolates with high-level ampicillin resistance evolved through a combination of single-point mutations in multiple genes involving efflux pumps, transcriptional regulators, and porins as well as by gene amplification increasingly focusing on the *ampC* locus. Deleting the *ampC* gene, we find that even in its absence, strains can still reach high-level resistance to ampicillin, by combining multiple mutations, often from the same pathways observed for the wild type.

We identified multiple genes under selection, and various resistance-conferring mutations within these genes. The combination of conducting multiple separate experiments and of sampling diverse isolates of varying resistance levels allowed for the identification of recurring mutations, a hallmark of positive selection. While some of these mutations appear in isolates selected for resistance levels which may be higher than encountered by bacteria in the clinic, some of these genes were already previously identified as resistance conferring in various settings. We assume that the adaptive pathways identified here do not represent an exhaustive catalogue of all available pathways. Further studies may be required to fully characterise the latent evolutionary potential, possibly with different increments of antibiotic concentration and varying plate dimensions. In addition, further study will be needed to understand the interaction, or epistasis, among single mutations within and between genes and how much they contribute to the resistance level, as well as the evolvability of different variants.

Our analysis of mutations together with the ampicillin resistance level observed for isolates carrying them, aimed at identifying dead-end mutations, pinpointed several genes such as envZ, rpoS, and ompC which had the characteristics expected from dead-end mutations. While we lacked the statistical power to conclusively identify these mutations as dead-end mutations, further experiments, for example, measuring the fitness cost of these mutations in relation to growth or mobility, may provide evidence for their apparently deficient propagation in the MEGA-plate setting. Additional experiments may also measure the fitness effect of these mutations in a multi-drug environment. It can be hypothesised that isolates carrying *ampC*-related mutations will have lower cross resistance towards other drugs than isolates carrying mutations in other genes, such as porins or efflux pumps, which are known to also confer resistance to other antibiotic drugs^23,46,47^.

Notably, we focused on the adaptability of a single chromosomally encoded beta-lactamase. While *ampC* is highly prevalent in human commensal bacteria such as *E. coli* and is often considered the basis for its clinical beta-lactam resistance^29,30^, additional beta-lactamases such as carbapenemase and cephalosporinase have an increasing clinical importance, especially in light of their rapid transfer via plasmids or other horizontally transferred elements. It will also be interesting to see what the evolutionary path to high-level ampicillin resistance is in different beta-lactamase classes and also in other, non-laboratory strain backgrounds and species, carrying these genes.

Despite these limitations, our results highlight the potential of the MEGA-plate in exploring multiple evolutionary pathways to high levels of resistance to beta-lactams. Critically, the identification of beta-lactamase-dependent and independent adaptive pathways to resistance may open the way towards pharmaceutically complementing beta-lactam treatment in the clinic such that common evolutionary paths to resistance are slowed down or inhibited.

## Methods

### Strains and media

The ancestral strains used in this study are *E. coli* BW25113 knockout strains ∆*lacA* (jw0333^48^, wild type ancestral) and ∆*ampC* (JW4111^48^, *ampC* deleted ancestral) from the Keio collection (carrying a kanamycin-resistant gene)^48^. For the longer experiments, Exps. C and D, these strains were further transformed with pZS2R plasmids coding a fluorescent marker (Yellow Fluorescent Protein, YFP) and a chloramphenicol resistance gene as an extra precaution to confirm that the samples we collected are descendant of the strains we inoculated. The media used in this study: BD Difco LB Agar (Lennox, 240110), BD Difco agar (Lennox, 214220) and BD Difco LB (Lennox, 240230). All antibiotics solutions were freshly prepared and filtered from powder stocks and used at the following concentrations: chloramphenicol (Sigma-Aldrich #C0378, 25 µg/ml), kanamycin (Sigma-Aldrich #K1377, 50 µg/ml), cycloheximide (Cyclo, Sigma #C7698, 100 µg/ml). Ampicillin (AMP, Sigma #A9518) was used at ranges of concentrations as indicated for each experiment (Supplementary Table 1).

### MEGA-plate construction

The base and outer walls of the MEGA-plates were built using a clear 6 mm thick polycarbonate sheet. Inner barriers and divisions, 1.5 cm high, were laser-cut from 3 mm thick plexiglass sheets. Components were welded using dichloromethane. The plate was covered with a heated glass cover to prevent condensation on the lid and provide optical clarity for imaging (Seaclear Industries, Electrically heated glass). The MEGA-plates were decontaminated before experiments with ~0.5% sodium hypochlorite (bleach) overnight and UV irradiation for between 30 min, and were decontaminated after the experiments with sodium hypochlorite. Experiments were performed in a temperature-controlled room at 30 °C and 70% humidity (Carel heaterSteam). The dimensions of the plate for each of the experiments are indicated in (Supplementary Table 1).

### MEGA-plate setup

The plate was composed of 3 agar layers. The bottom and the middle agar layers were made of LB Agar sterilised by autoclaving in a 20-min cycle. Further, to avoid contamination, media was supplemented with kanamycin and cycloheximide. To maintain fluorescence encoding plasmid, media was also supplemented with chloramphenicol. To improve imaging, 5 ml of autoclaved India Ink (Higgins #44204) was added to each liter. An increasing concentration of ampicillin was added to the bottom layer to make the gradient of ampicillin concentrations. Once the black agar has solidified, the middle layer, a black agar without ampicillin but with kanamycin, chloramphenicol (in case the plasmid was present), and cycloheximide was poured on top of the bottom layer and was let to solidify. The addition of a thin middle layer (0.1 cm height) of solid agar between the bottom (1.4 cm height) and overlay decreases the inhomogeneity of growth between sections by physically levelling the interface. Once these layers solidified, we poured on top the soft agar layer (0.3 cm height), composed of 0.2% w/v agar with 2% w/v LB and supplemented with kanamycin, cycloheximide, and chloramphenicol (experiment C and experiment D). The ancestral strain was then inoculated at 3 µl (~10^6^ cells) at the ampicillin-free well of the plate.

### MEGA-plate Imaging

For time-lapse photography, images of the MEGA-plate were taken at constant intervals (as indicated below) with a Canon T3i controlled by digiCamControl software. The black ink added to the bottom and middle agar layers, combined with white-light LED strip lights at an indirect angle allows to obtain a high-contrast image across the plate and avoid reflection. Images were aligned, cropped, and linearly contrast-enhanced in each colour channel. To remove illumination differences across the plate, the first image of each experiment, taken before inoculation, was subtracted from all following images in each colour channel. All enhancements were done in MATLAB and are uniform across all regions of all images in the same experiment. Movies were constructed from the processed images using MATLAB (v. R2021a).

### Collecting resistant clones across the MEGA-plate

Once bacteria spread, evolved, and reached the highest antibiotic concentration region, the experiment was terminated and different spots of the MEGA-plate were sampled according to the evolutionary histories of the evolved bacteria as determined by the time-lapse movie (Fig. 1c and Supplementary Fig. 1; Exp B and E was stopped earlier due to fungus contamination). From each indicated location across the MEGA-plate (Supplementary Fig. 1), 1 µl of the swim agar was collected into 50 µl PBS in 96-well plates and stored in glycerol stock at −80 ^°^C. These collected samples were then spread on LB agar plates (without ampicillin, but with KAN, and CHL for Exp C, D), and independent colonies were collected (3 colonies per sample for Ex. A, C, and D; 1 colony for B and E).

### Measuring bacterial advancement on the MEGA-plate

To analyse the progression of bacteria on the MEGA-plate, we used a custom code measuring the intensity in the sample collection spots over time. For each spot and time point, we calculated the mean intensity of the area (9 × 9 pixels, Supplementary Fig. 1) in the red channel. Then subtracted the first image and smoothed the progression over time by taking a 7-frame moving mean of these intensity values. By choosing a threshold of >3 (on a scale of 0 to 255), we found the first time point in which bacteria advanced to each sampling location.

### High-throughput minimal inhibitory concentration (MIC) measurement

To determine the MIC of each sample taken from across the MEGA-plate, the collected colonies were inoculated on a series of plates containing increasing ampicillin concentration and supplemented with KAN and CHL and incubated for 48 h at 30 °C. After incubation, images of the plates were taken and automated computer analysis was used to identify the lowest concentration at which no growth occurred. Pixels were binarized (black/white) and growth was called if more than 2% of the area was white (indicated by cyan contour, Supplementary Datasets 1–4) meaning isolates with sporadic and inconsistent growth on the ampicillin gradient were excluded from later analysis. Then MIC was determined by growth-area weighted interpolation of the antibiotic concentrations of two plates - the highest concentration with growth on it and the one above it. MIC is indicated as an orange line drawn on a bar of concatenated images per isolate.

### DNA extraction and sequencing library preparation

Colonies were grown overnight in antibiotic-free LB broth. Overnight cultures were centrifuged and DNA was extracted by NucleoSpin 96 Tissue core kit (Macherey-Nagel). Extracted DNA concentration was measured by Quant-iT kit (Thermo Fisher Scientific) and standardised to 1.5 ng/ul. Illumina sequencing libraries were prepared as previously described^49^. Briefly, standardised DNA was tagmented (Illumina kits FC-121-1030 or FC-121-1031). Tagmented libraries were indexed and amplified by the KAPA HiFi Library Amplification kit (KAPA KK2611/KK2612). Size selection and clean-up of libraries were done using 0.8 volumes of AMPure beads (Beckman Coulter).

### Whole-genome sequencing and analysis

Libraries were sequenced in Rapid-mode on a HiSeq 2500 Illumina machine to produce 125 bases long paired-end reads. Illumina reads were filtered to remove reads contaminated by the Nextera adapter or low-quality bases (>2 bases with a Phred Score of <20), yielding an average of 1.4 M reads per sample (s.d. = 0.81 M). These reads were aligned to the reference genome of *E. coli* BW25113 (Accession number: CP009273) using Bowtie 1.2.1.1, while allowing a maximum of 3 mismatches per read. Base-calling was done using SAMtools and BCFtools 0.1.19. A genome position was identified as an SNP separating between isolates if more than a single allele was identified across isolates from a single experiment using a quality threshold of FQ < − 80.

### Nucleotide copy number analysis

For each isolate, a ‘raw copy number’ for each nucleotide was calculated as the base coverage divided by the median coverage of the genome of the isolate in the covered bases (where coverage > 0).

### Gene copy number analysis

For each isolate, a ‘raw copy number’ for each gene was calculated as the median base coverage across the gene divided by the median coverage of called positions across the genome of the isolate. To remove gene-specific biases and determine copy number, this raw copy number was further normalised by the median raw copy number value of the gene across all sequenced unevolved isolates (either from the bacterial stock used to inoculate the MEGA-plate or isolates collected from the ampicillin-free well on the MEGA-plate), resulting in a normalised gene copy number.

### SNP analysis

For each gene, the number of mutational events is derived from all observations of SNPs in that gene across experiments by counting each distinct mutation only once per each experiment in which it appears. To identify genes enriched for mutations, we compared the distribution of the number of mutational-events-per-gene with the random expected distribution as identified by simulation in which the same total number of identified SNPs per strain (wt or *ΔampC*) were given random positions across the genome (Supplementary Fig. 4). To identify dead-end mutations, we looked for mutations which appeared associated with a single MIC in the experiment(s) in which they were observed. In an experimental system where parallel lineages are sampled multiple times and isolates of differing MICs are observed, mutations being associated with a single MIC may suggest a dead-end genotype. Therefore, we counted, for each gene the number of mutational events which were only observed in isolates sharing a single MIC. To test whether this number is more than expected, we measured the chances of observing this number (or higher) in simulated data in which mutations were shuffled between different isolates in the same experiment. The chances were further divided by the number of considered genes to calculate a multiple-hypothesis corrected p-value.

### Serial passage evolution experiment

Experimental evolution was performed in liquid LB medium based on a previously established evolution experiment^50^ for approximately 90 generations (9 transfers). The ancestral wild type strain was inoculated (~10^6^ cells) into each well containing fresh medium with increasing dosage of ampicillin (0.25X, 0.5X, 1X, 2X, 4X, 8X, 16X, 32X, 64X, 128X, 256X, 512X the MIC) and 50 µg/ml of KAN to prevent contamination. After 21-h growth, the optical density at 600 nm (OD_600_) of each culture was measured in a BioTek PowerWave 340 plate reader, and the culture from the highest antibiotic concentration in which bacteria grew (OD_600_ > 0.1) was diluted by 1/1000 and was used to inoculate a freshly made gradient of ampicillin. This serial passage evolution experiment was performed in three different volumes, corresponding to different population sizes: (a) low: 120 µl, 10^8^ cells; (b) medium: 600 µl, ~5 * 10^8^ cells; (c) high: 8 ml, ~10^10^ cells. All the experiments with low, medium, and high volumes included seven parallel replicates. To validate the efficacy of the drug, the same gradient of ampicillin was also inoculated with the wild type strain in each of the daily transfers (showing variations of less than a factor of 2 among days, Supplementary Fig. 5, ancestors).

### Reporting summary

Further information on research design is available in the Nature Portfolio Reporting Summary linked to this article.

### Supplementary information

Supplementary information

Peer Review File

Description of Additional Supplementary Files

Supplementary Dataset 1

Supplementary Dataset 2

Supplementary Dataset 3

Supplementary Dataset 4

Supplementary Dataset 5

Supplementary Dataset 6

Reporting Summary

### Source data

Source data

## Data Availability

The whole-genome sequencing data generated in this study have been deposited in the NCBI SRA database under accession code PRJNA926794. Raw images as well as time-lapse movies of all MEGA-plate experiments have been deposited in Zenodo under 10.5281/zenodo.7950385. Source data are provided with this paper.

## Code availability

Custom code developed in this study are deposited in Zenodo under 10.5281/zenodo.7950385.
