# Supplementary material for: Beta-lactamase dependent and independent evolutionary paths to high-level ampicillin resistance
Source: Nat Commun. 2024 Jun 25;15:5383. doi: 10.1038/s41467-024-49621-2 (PMC11199616; doi:10.1038/s41467-024-49621-2)
Supplement: Supplementary file 1 — Supplementary information [file 41467_2024_49621_MOESM1_ESM.pdf]

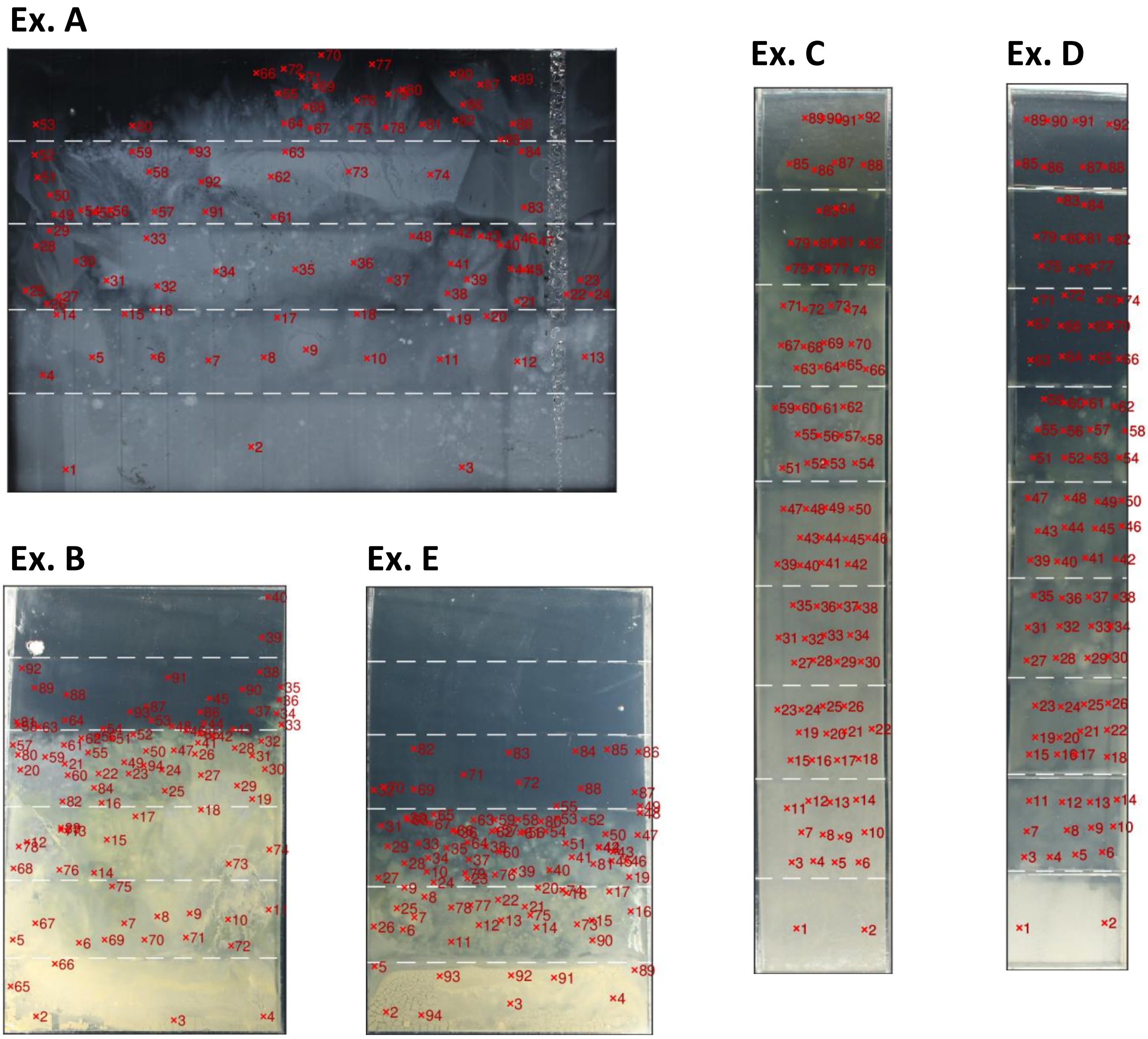


**Supplementary Figure 1. Bacteria evolved on ampicillin MEGA-plate and were sampled.** The last-time point from the movie with the area of sampling. Each X represents an area on the MEGA-plate which was sampled. The white dashed lines represent the barriers between sections. **a,** WT bacteria evolved for ~9.5 days. **b,** WT bacteria evolved for ~9.5 days. **c,** Δ*ampC* bacteria evolved for ~9.5 days. **d,** WT bacteria evolved for ~30 days. **e,** Δ*ampC* bacteria evolved for ~30 days. (See supplementary table 1 for more details). Source data are provided as a Source Data file.

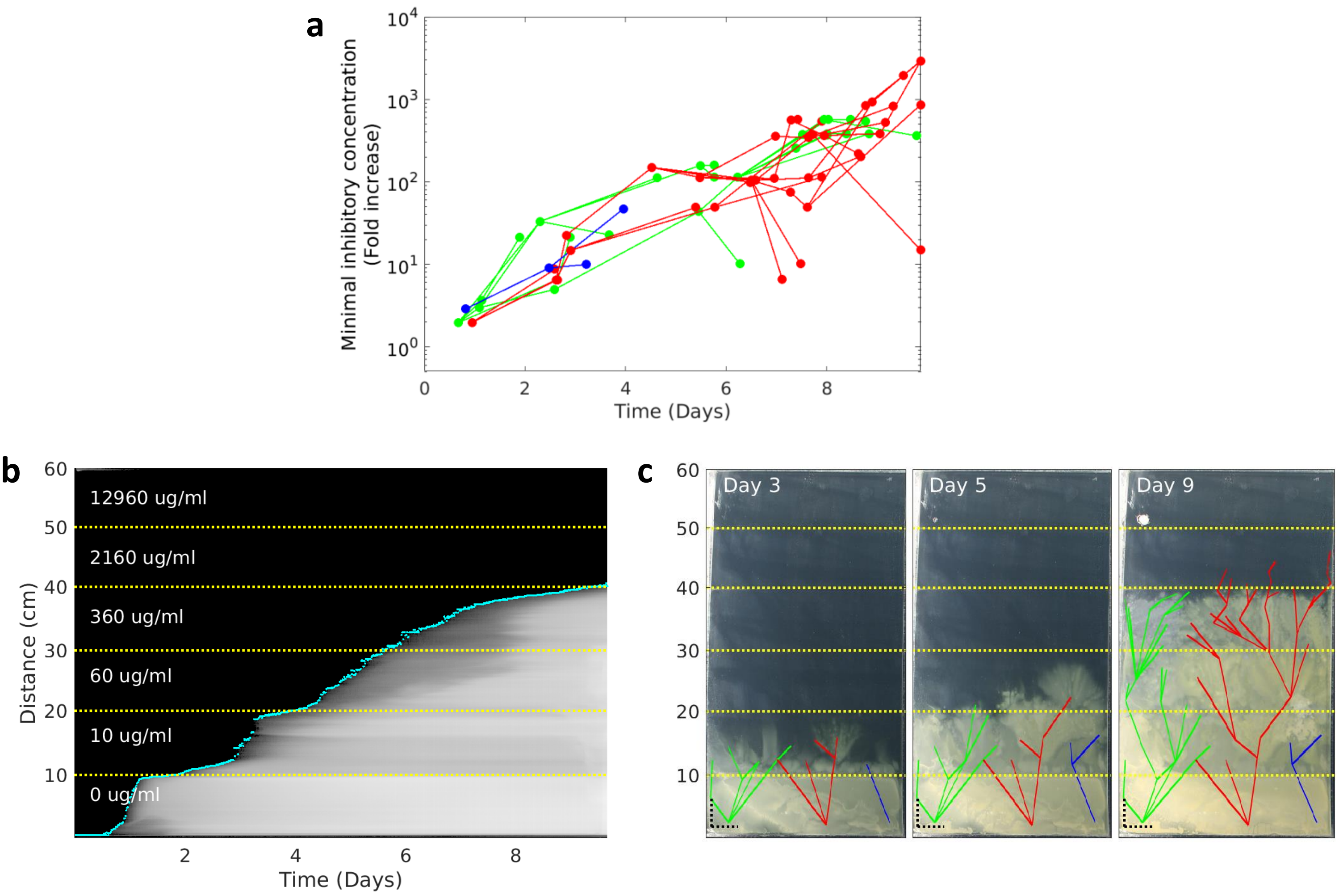


**Supplementary Figure 2. MEGA-plate experimental setting allows evolution of high-level ampicillin resistance.**
**a,** The increase in resistance levels as a function of time for isolates from three distinct lineages (green, red, blue) of the MEGA-plate. **b,** A kymograph image of the MEGA-plate experiment showing for each time-point the horizontally 90 top percentile of the averaged pixel intensity of the plate. The cyan dots represent the farthest point reached by the bacteria at each time point. **c,** Images of the MEGA-plate at three-time points (day 3,5,9), indicating the locations of isolates sampled from three distinct lineages (red, green, and blue connected points). Images are shown with an aspect ratio of 1:1, as indicated by the horizontal and vertical dashed black scale, both representing 5 cm. In both b and c, concentration steps are indicated by yellow dashed lines. Source data are provided as a Source Data file.

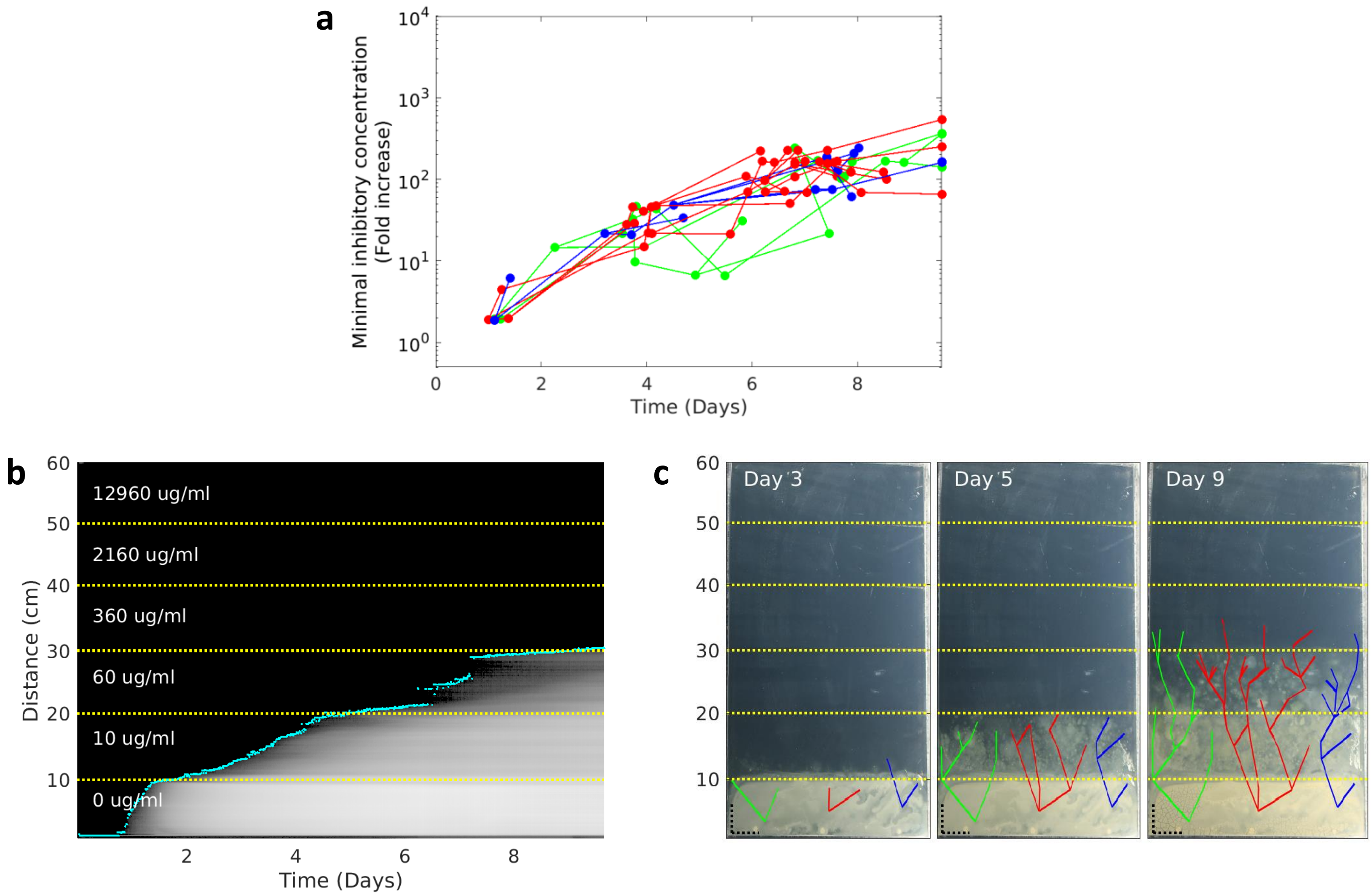


**Supplementary Figure 3. MEGA-plate experimental setting allows the evolution of high-level ampicillin resistance.**

**a,** The increase in resistance levels as a function of time for isolates from three distinct lineages (green, red, blue) of the MEGA-plate. **b,** A kymograph image of the MEGA-plate experiment showing for each time-point the horizontally 90 top percentile of the averaged pixel intensity of the plate. The cyan dots represent the farthest point reached by the bacteria at each time point. **c,** Images of the MEGA-plate at three-time points (day 3,5,9, indicated by vertical black-white lines in c), indicating the locations of isolated sampling from three distinct lineages (red, green, and blue connected points). Images are shown with an aspect ratio of 1:1, as indicated by the horizontal and vertical dashed black scale, both representing 5 cm. In both b and c, concentration steps are indicated by yellow dashed lines. Source data are provided as a Source Data file.

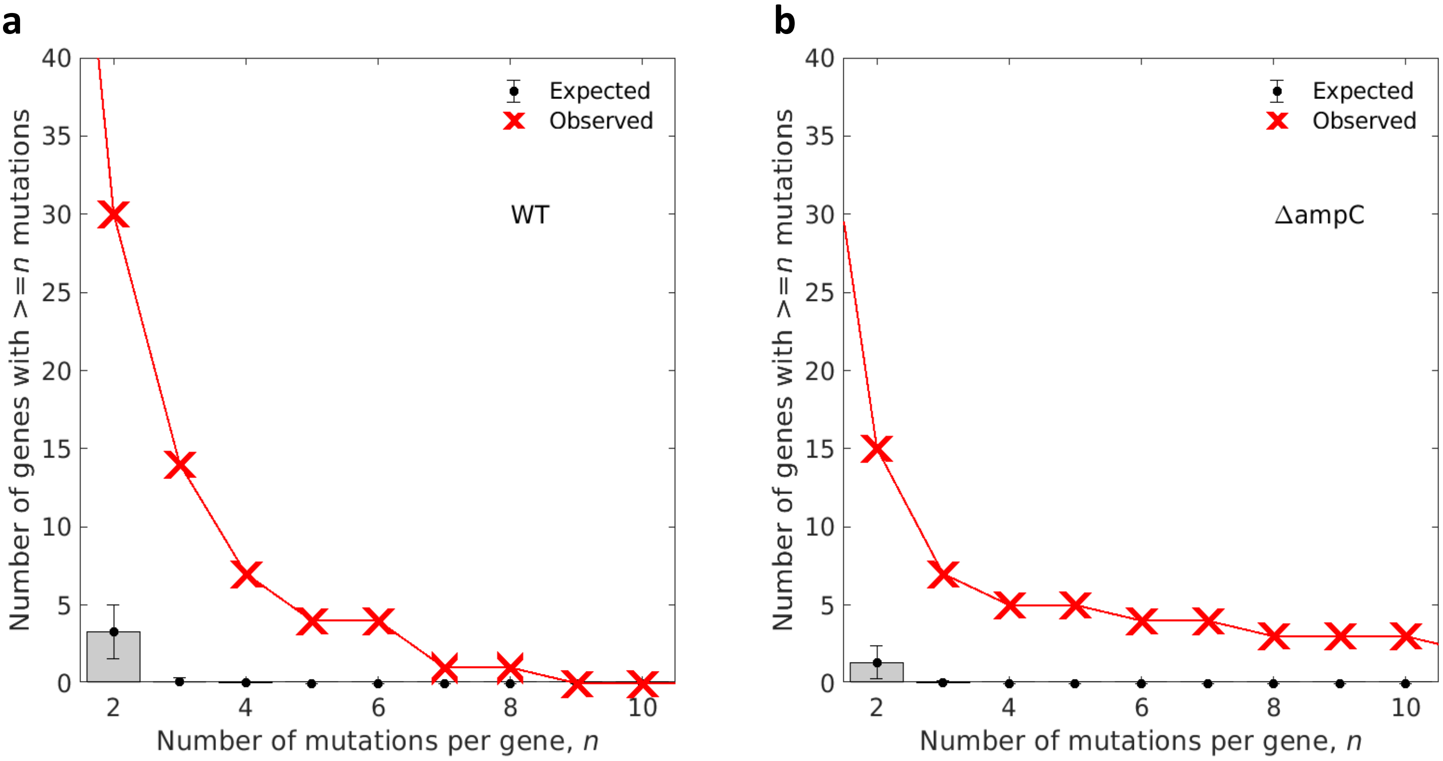


**Supplementary Figure 4. Comparison of the distribution of the number of events-per-gene with the random distribution as identified by simulation.**

The number of events-per-gene counts also the same SNP in parallel experiments. The analysis takes in consideration the number of genes with the same numbers of events-per-gene and plots it (red line and X). The simulation took in consideration the total number of SNPs and 1000 times randomly spread them in the genome and then counted the number of genes with the numbers of events per gene (gray bars and black dots). **a,** wild type . **b,** *ampC* deleted. Source data are provided as a Source Data file.

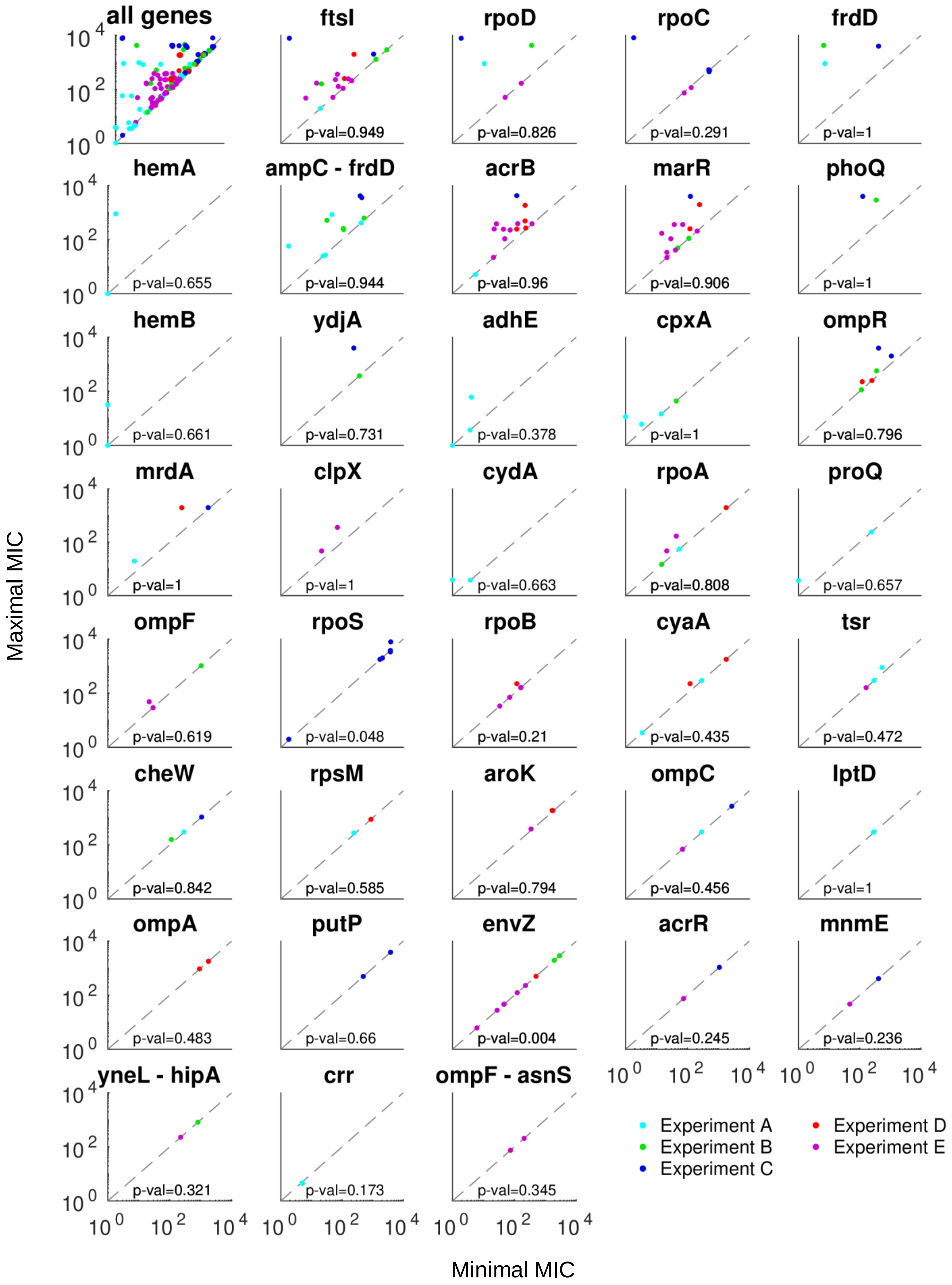


**Supplementary Figure 5. Mutational event MIC range.**
A mutational event is a specific SNP observed in one or more isolates in a single experiment (plate). For each SNP enriched gene, mutational events are plotted according to the minimal and maximal MIC of the isolate(s) in which they were observed in their respective experiments. P-values corrected for multiple hypothesis (number of genes) are calculated by comparing to simulated shuffled set of mutations (Methods).

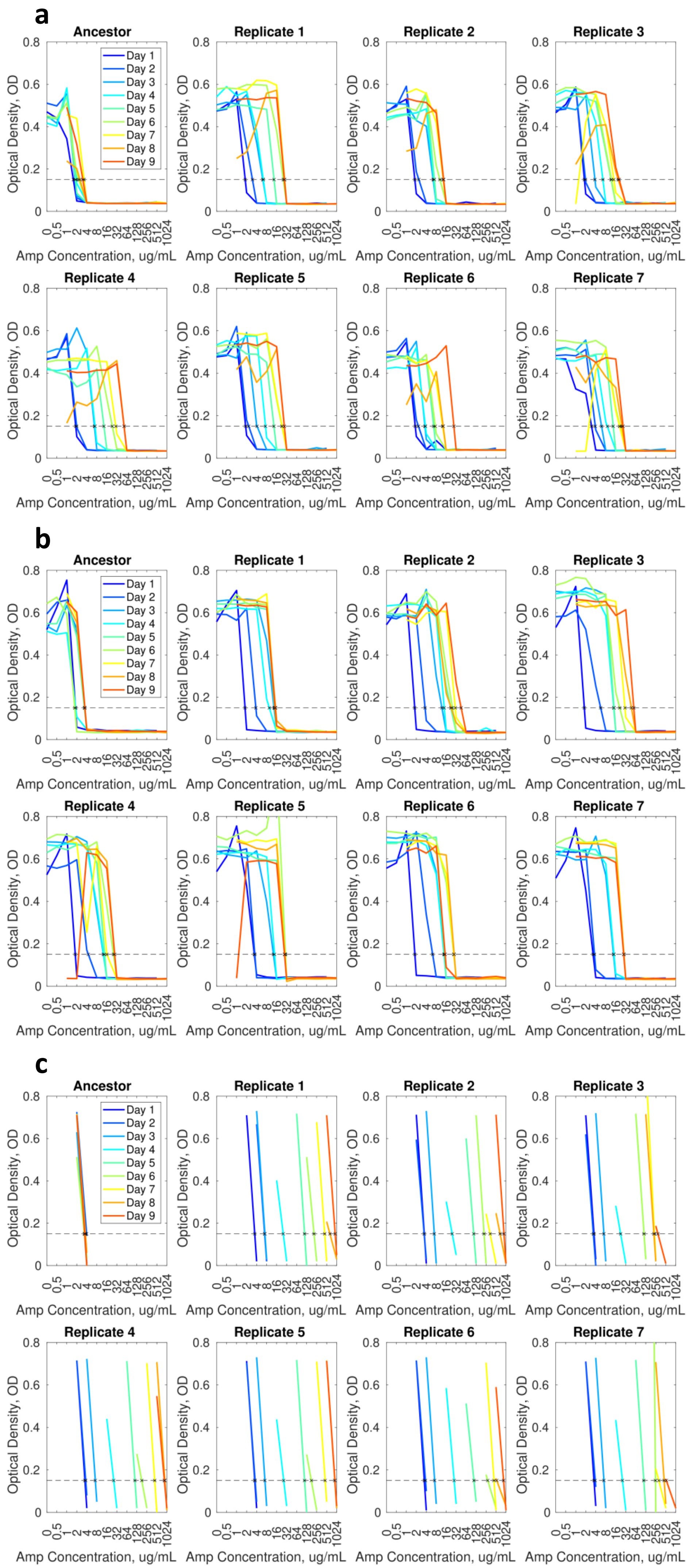


**Supplementary Figure 6. OD measurements for serial passage evolution experiment.** The optical density of each tube was measured at 600 nm ($OD_{600}$) in a BioTek PowerWave 340 plate reader every 21 hours before passage. The well with highest ampicillin concentration showing $OD_{600}$>0.1 was diluted 1/1000 and transferred to a new set of tubes with an ampicillin gradient. Each tube contained LB broth with an ampicillin concentration two times higher than the one before, at the range of 0.5-1024 µg/ml ampicillin. All tubes contained 50 µg/ml of KAN to prevent contamination. **a**, low-volume (120 µl). **b,** medium-volume (600 µl). **c,** large volume 8 ml. Source data are provided as a Source Data file.

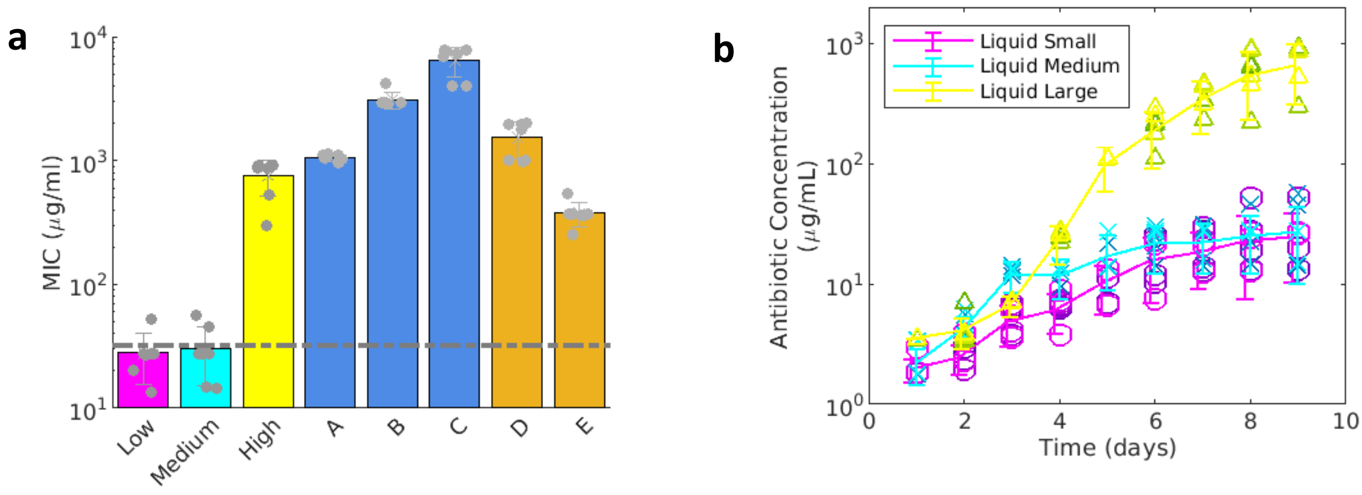


**Supplementary Figure 7. MIC dynamics across different laboratory evolution experiments.** **a,** The bars show the mean MICs of the seven most resistant from each experiment (n=7 isolates or wells). The gray dots represent the measured MIC of each one of the isolates with the standard error of the mean. The colors show the type of the experiment: Magenta is a 120µl liquid experiment, cyan is a 600µl liquid experiment, yellow is 8ml liquid experiment, blue is MEGA-plate experiment inoculated with the wild type ancestral strain and orange is MEGA-plate experiment inoculated with the *ampC* deleted strain. **b,** Serial passage evolution experiment was performed in three different volumes, corresponding to different population sizes (low: 120µl, $10^8$ cells, magenta; medium: 600µl, ~$5*10^8$ cells, cyan; high: 8ml, ~$10^{10}$ cells, yellow). The gray dashed line in a and b is the clinical breakpoint. Source data are provided as a Source Data file.

| Exp | Strain | Width X Length | Concentrations | Time |
|---|---|---|---|---|
| A | WT | 80cmX60cm | 0, 4.8, 24, 120, 600 μg/ml | 9.51 days |
| B | WT | 37.5cmX60cm | 0, 10, 60, 360, 2160, 12960 μg/ml | 9.46 days |
| C | WT | 10cmX80cm | 0, 10, 35, 122.5, 428.75, 1500, 5252, 18382, 64339 μg/ml | 29.91 days |
| D | ΔampC | 10cmX80cm | 0, 10, 35, 122.5, 428.75, 1500, 5252, 18382, 64339 μg/ml | 29.91 days |
| E | ΔampC | 37.5cmX60cm | 0, 10, 60, 360, 2160, 12960 μg/ml | 9.46 days |

**Supplementary Table 1**
