# Supplementary material for: Beta-lactamase dependent and independent evolutionary paths to high-level ampicillin resistance
Source: Nat Commun. 2024 Jun 25;15:5383. doi: 10.1038/s41467-024-49621-2 (PMC11199616; doi:10.1038/s41467-024-49621-2)
Supplement: Supplementary file 3 — Description of Additional Supplementary Files [file 41467_2024_49621_MOESM3_ESM.pdf]

Description of Additional Supplementary Files:

Supplementary Dataset 1: Ampicillin resistance measurements for isolates collected in Experiment A. Bacteria inoculated on a series of agar plates with increasing ampicillin concentration and growth measured by image analysis after overnight incubation (Methods). Cyan lines represent the boundaries of detected growth areas. Interpolated MIC is indicated as an orange line.

Supplementary Datasets 2: Ampicillin resistance measurements for isolates collected in Experiment B. Bacteria inoculated on a series of agar plates with increasing ampicillin concentration and growth measured by image analysis after overnight incubation (Methods). Cyan lines represent the boundaries of detected growth areas. Interpolated MIC is indicated as an orange line.

Supplementary Datasets 3: Ampicillin resistance measurements for isolates collected in Experiment C and D. Bacteria inoculated on a series of agar plates with increasing ampicillin concentration and growth measured by image analysis after overnight incubation (Methods). Cyan lines represent the boundaries of detected growth areas. Interpolated MIC is indicated as an orange line.

Supplementary Datasets 4: Ampicillin resistance measurements for isolates collected in Experiment E. Bacteria inoculated on a series of agar plates with increasing ampicillin concentration and growth measured by image analysis after overnight incubation (Methods). Cyan lines represent the boundaries of detected growth areas. Interpolated MIC is indicated as an orange line.

Supplementary Dataset 5: Annotation of mutations identified across MEGA-plate experiments in the wt strain. Mutations are positions in the reference genome for which more than a single allele was identified across isolates. Base calls for all isolates are included as either A,C,G,T,N or n, where N indicates no coverage and n indicates low call quality.

Supplementary Dataset 6: Annotation of mutations identified across MEGA-plate experiments in the delta-ampC strain. Mutations are positions in the reference genome for which more than a single allele was identified across isolates. Base calls for all isolates are included as either A,C,G,T,N or n, where N indicates no coverage and n indicates low call quality.
