# Supplementary material for: Beta-lactamase dependent and independent evolutionary paths to high-level ampicillin resistance
Source: Nat Commun. 2024 Jun 25;15:5383. doi: 10.1038/s41467-024-49621-2 (PMC11199616; doi:10.1038/s41467-024-49621-2)
Supplement: Supplementary file 4 — Supplementary Dataset 1 [file 41467_2024_49621_MOESM4_ESM.pdf]

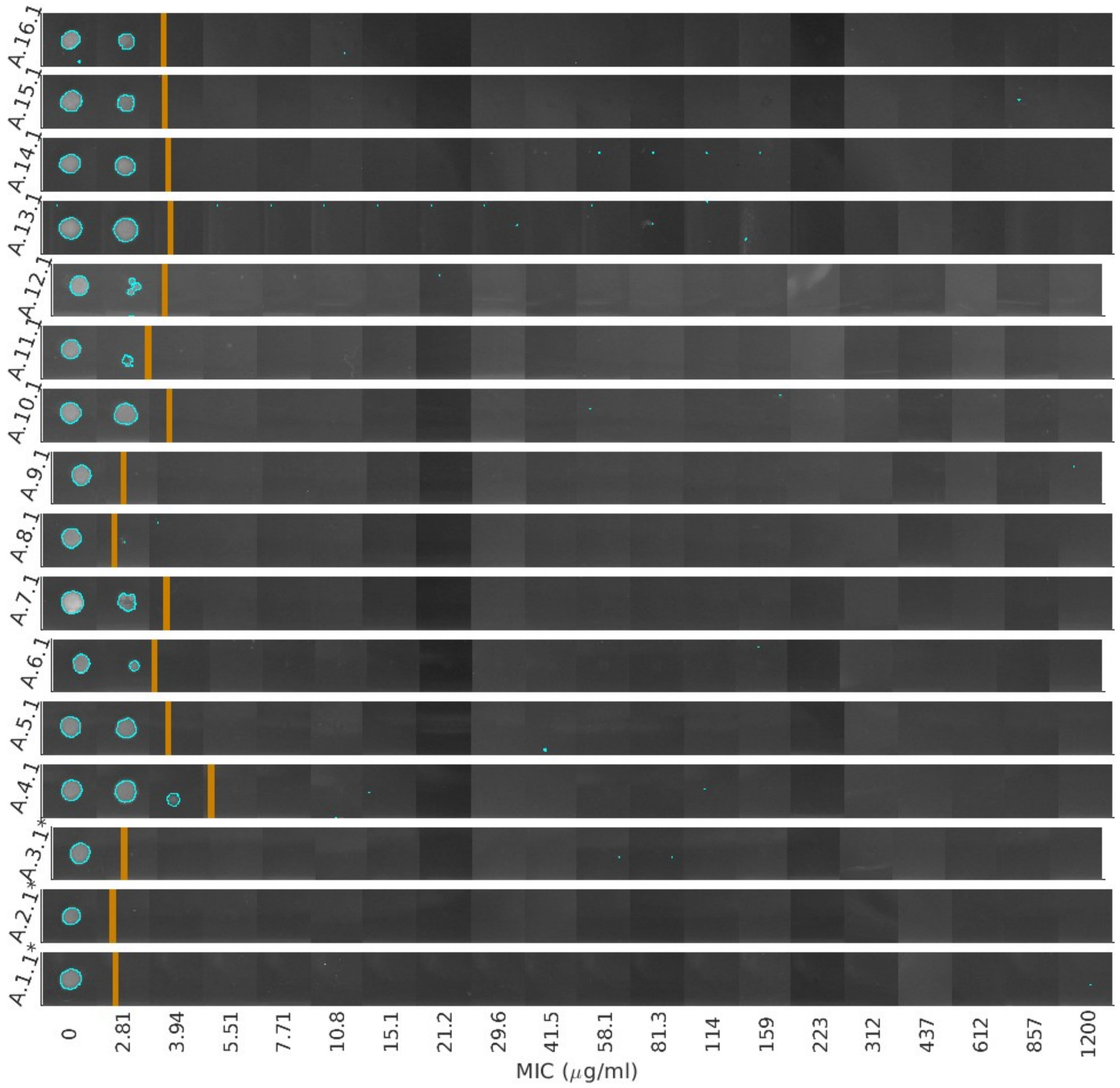

Supplementary Datasets 1: Ampicillin resistance measurements for isolates collected in Experiment A. Bacteria inoculated on a series of agar plates with increasing ampicillin concentration and growth measured by image analysis after overnight incubation (Methods). Cyan lines represent the boundaries of detected growth areas. Interpolated MIC is indicated as an orange line. 1/36

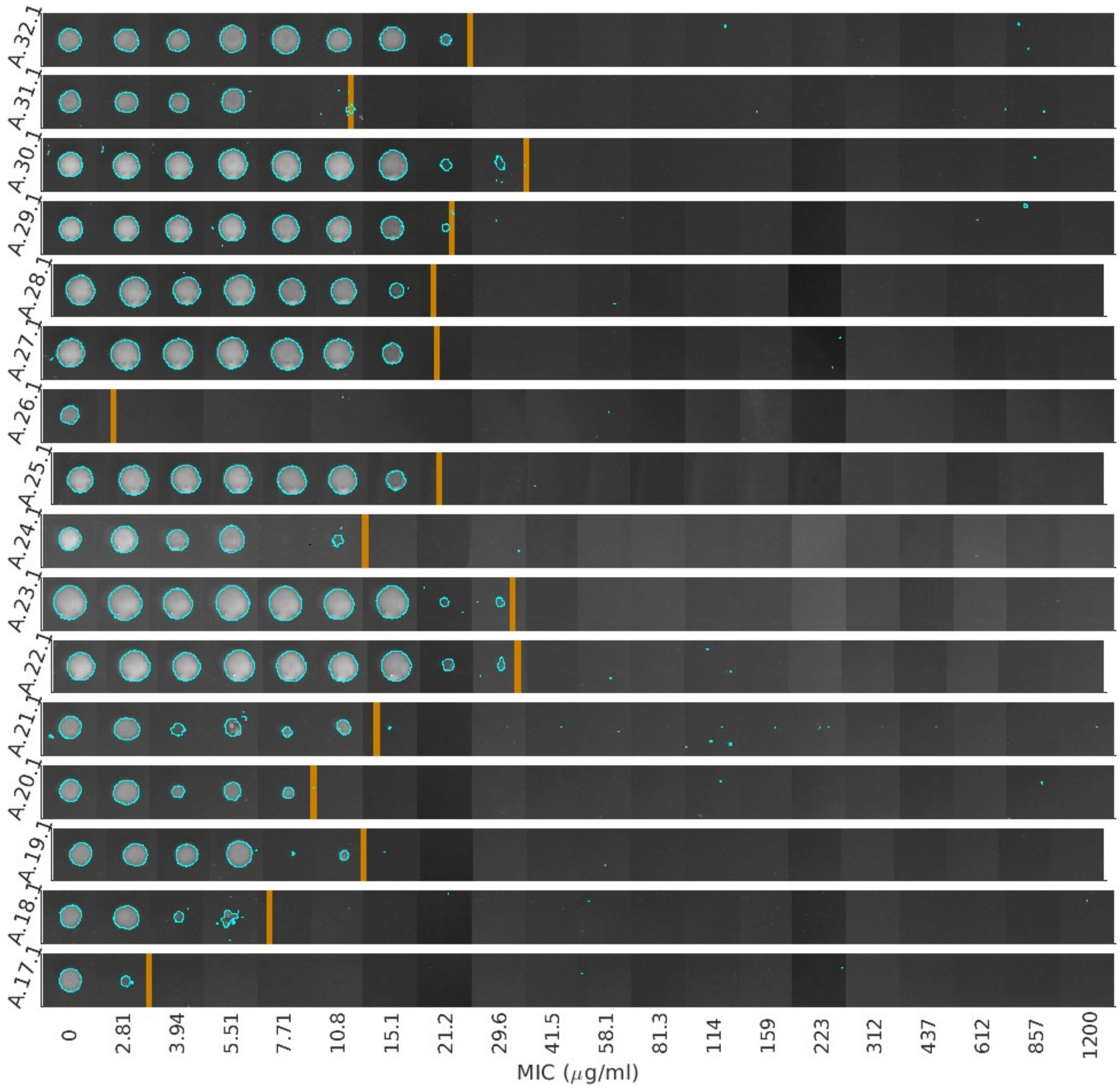

Supplementary Datasets 1: Ampicillin resistance measurements for isolates collected in Experiment A. 2/36

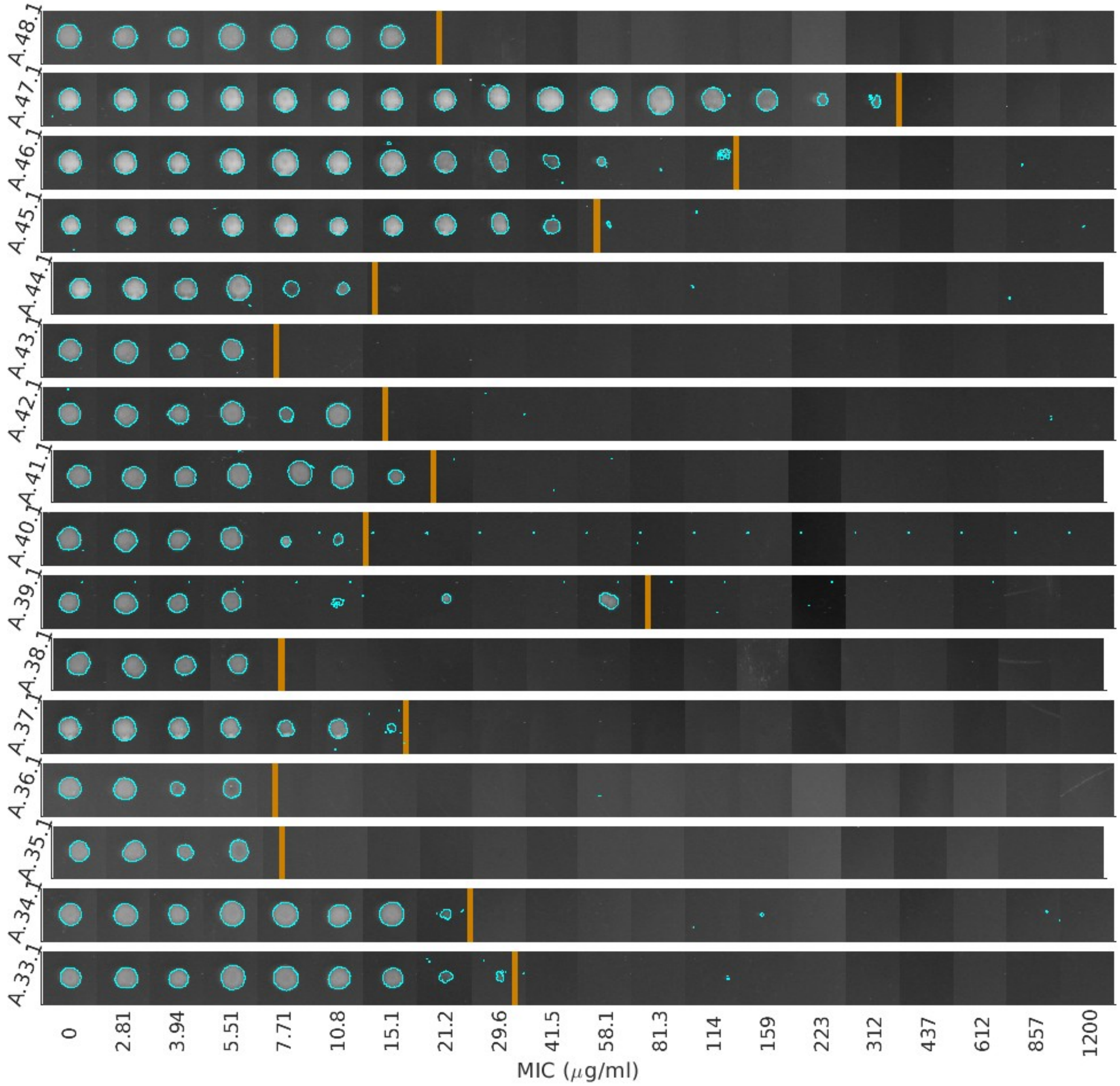

Supplementary Datasets 1: Ampicillin resistance measurements for isolates collected in Experiment A.

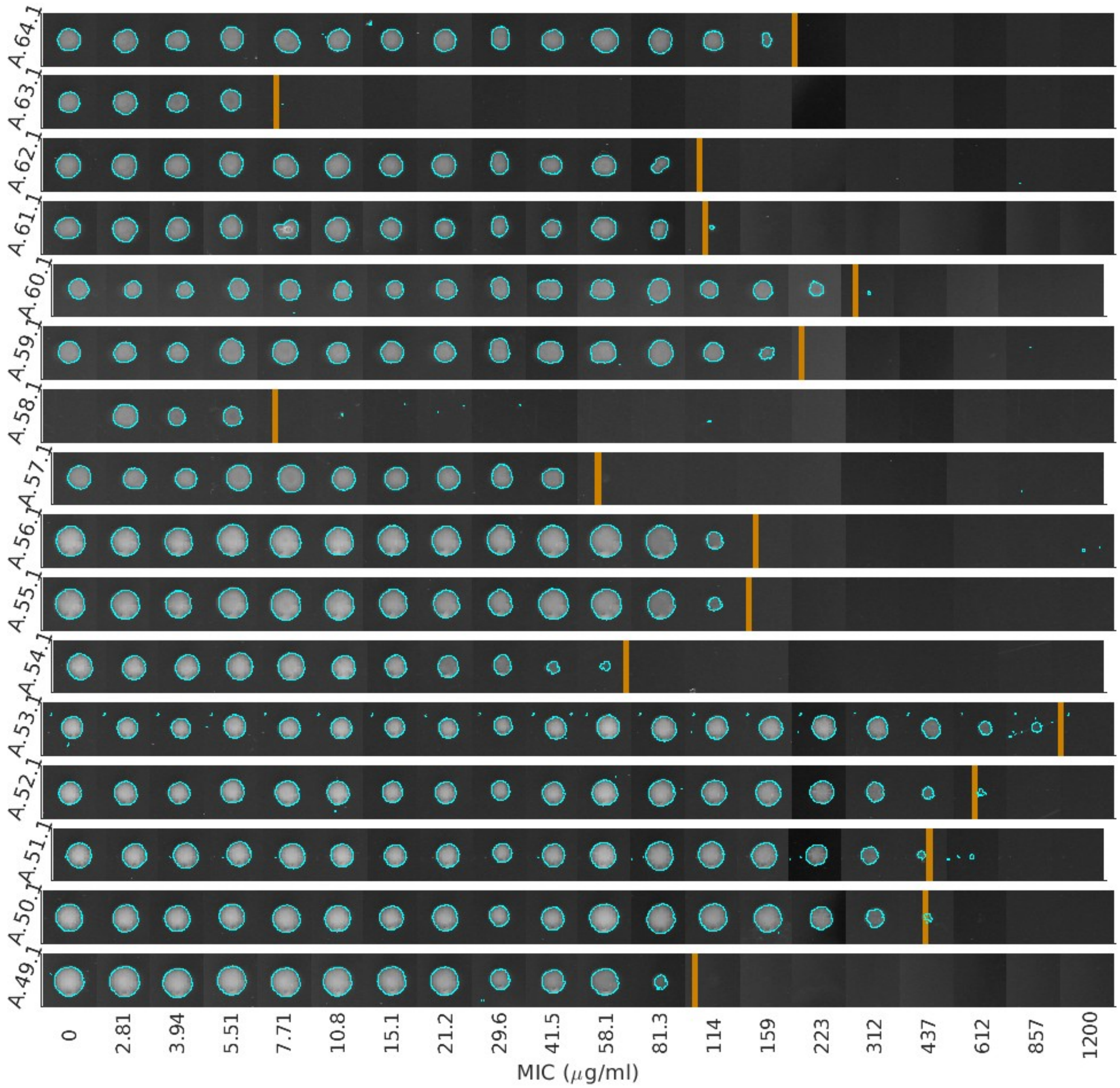

Supplementary Datasets 1: Ampicillin resistance measurements for isolates collected in Experiment A.

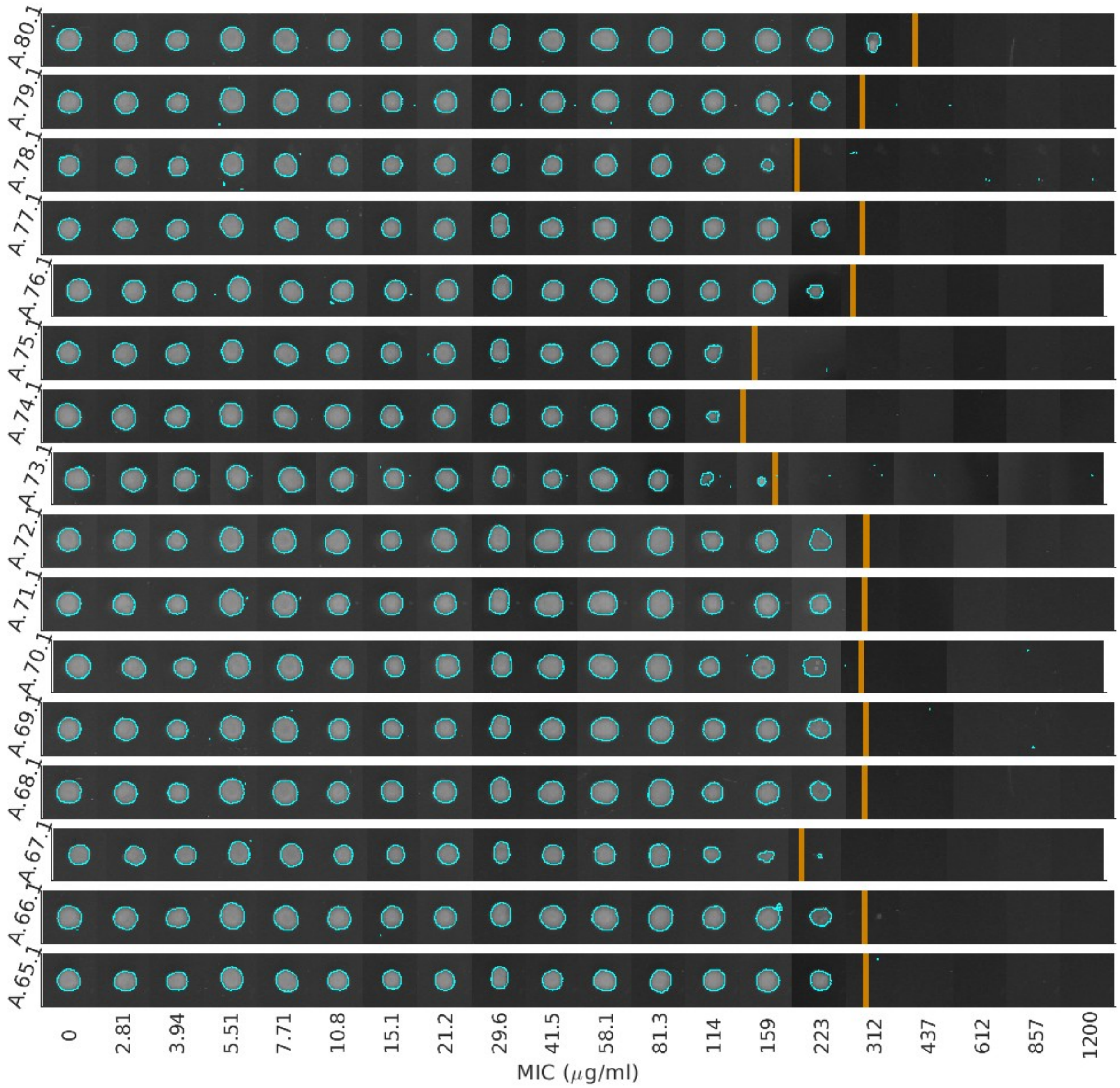

Supplementary Datasets 1: Ampicillin resistance measurements for isolates collected in Experiment A. 5/36

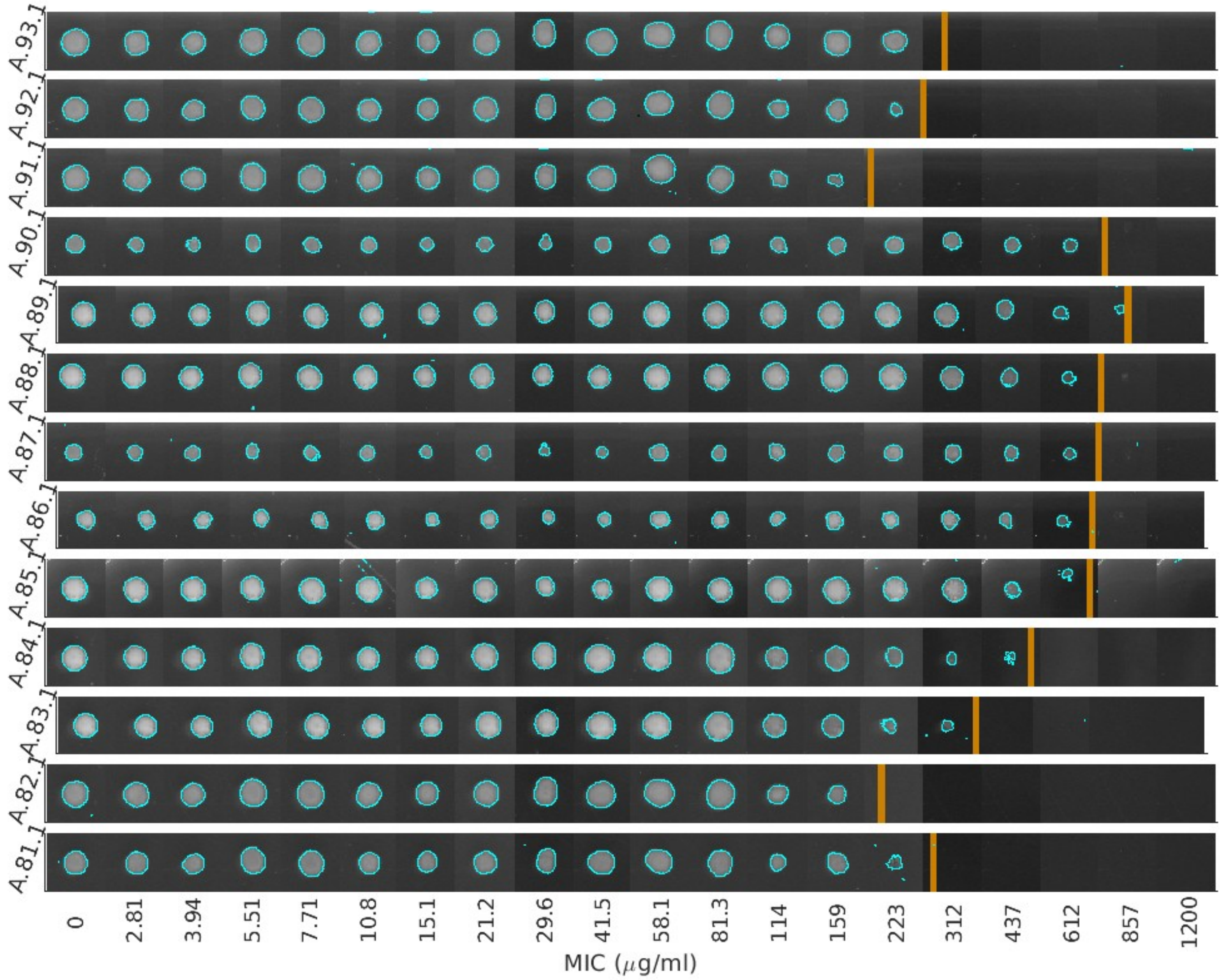

Supplementary Datasets 1: Ampicillin resistance measurements for isolates collected in Experiment A.

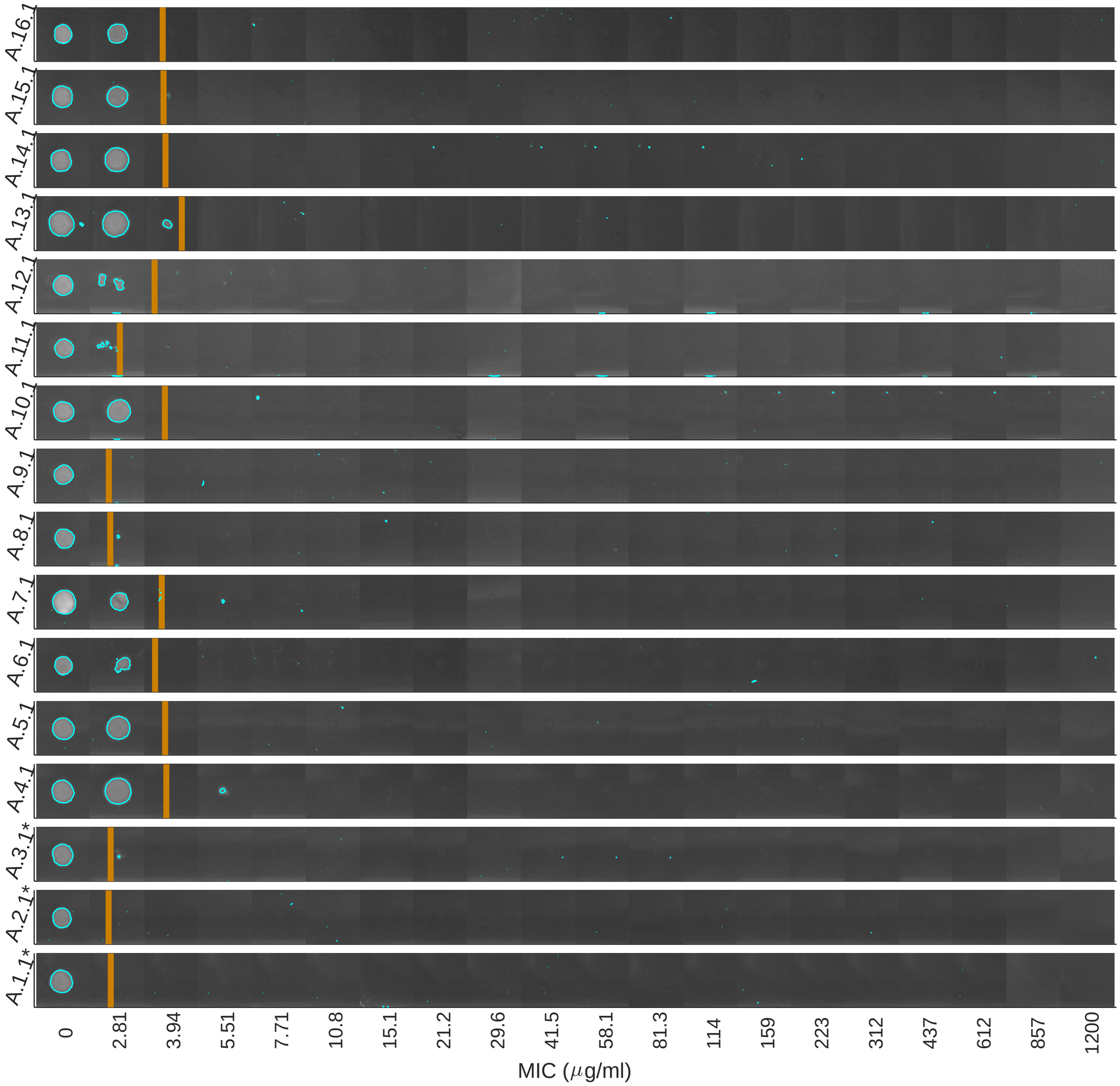

Supplementary Datasets 1: Ampicillin resistance measurements for isolates collected in Experiment A.

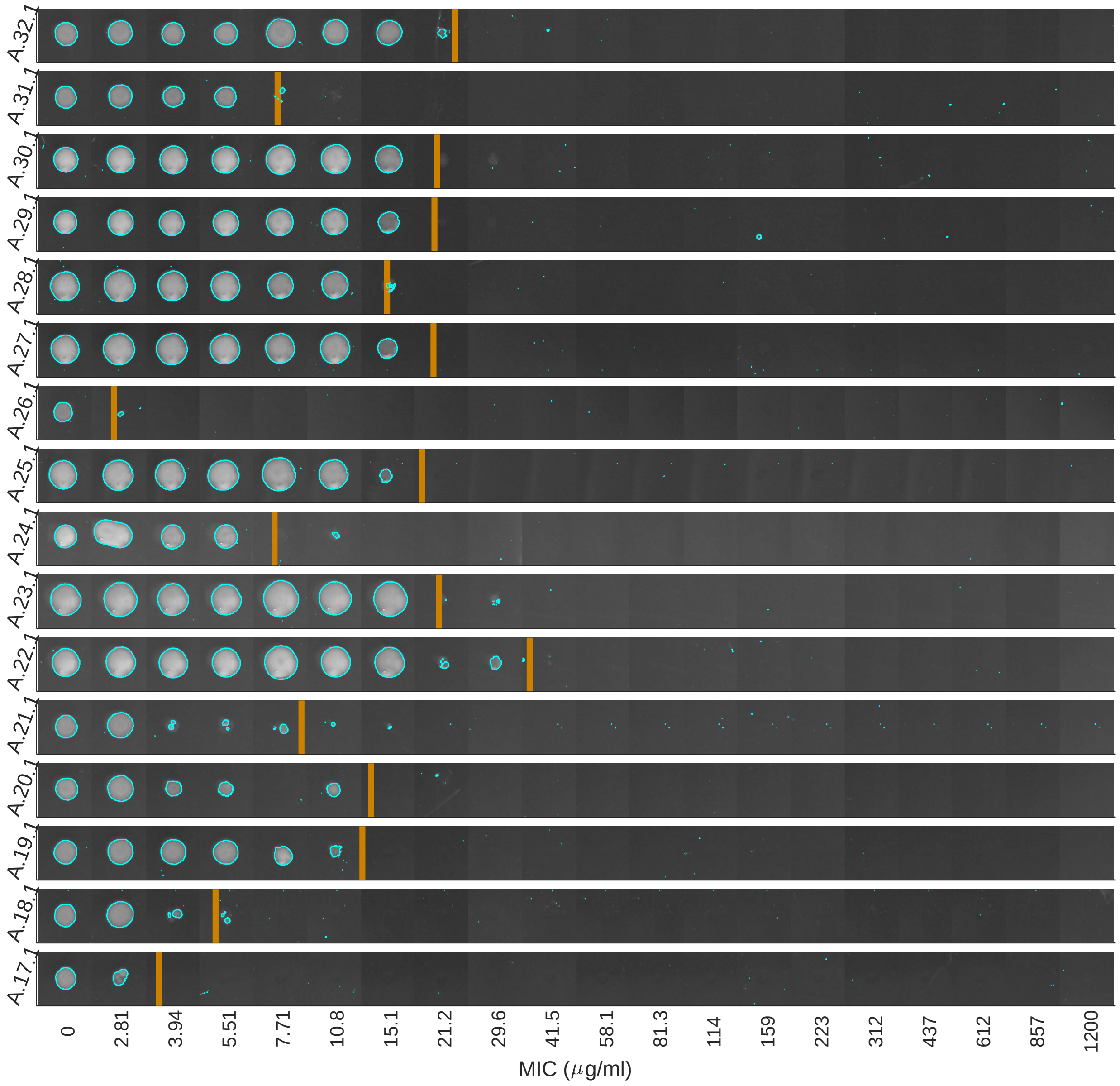

Supplementary Datasets 1: Ampicillin resistance measurements for isolates collected in Experiment A.

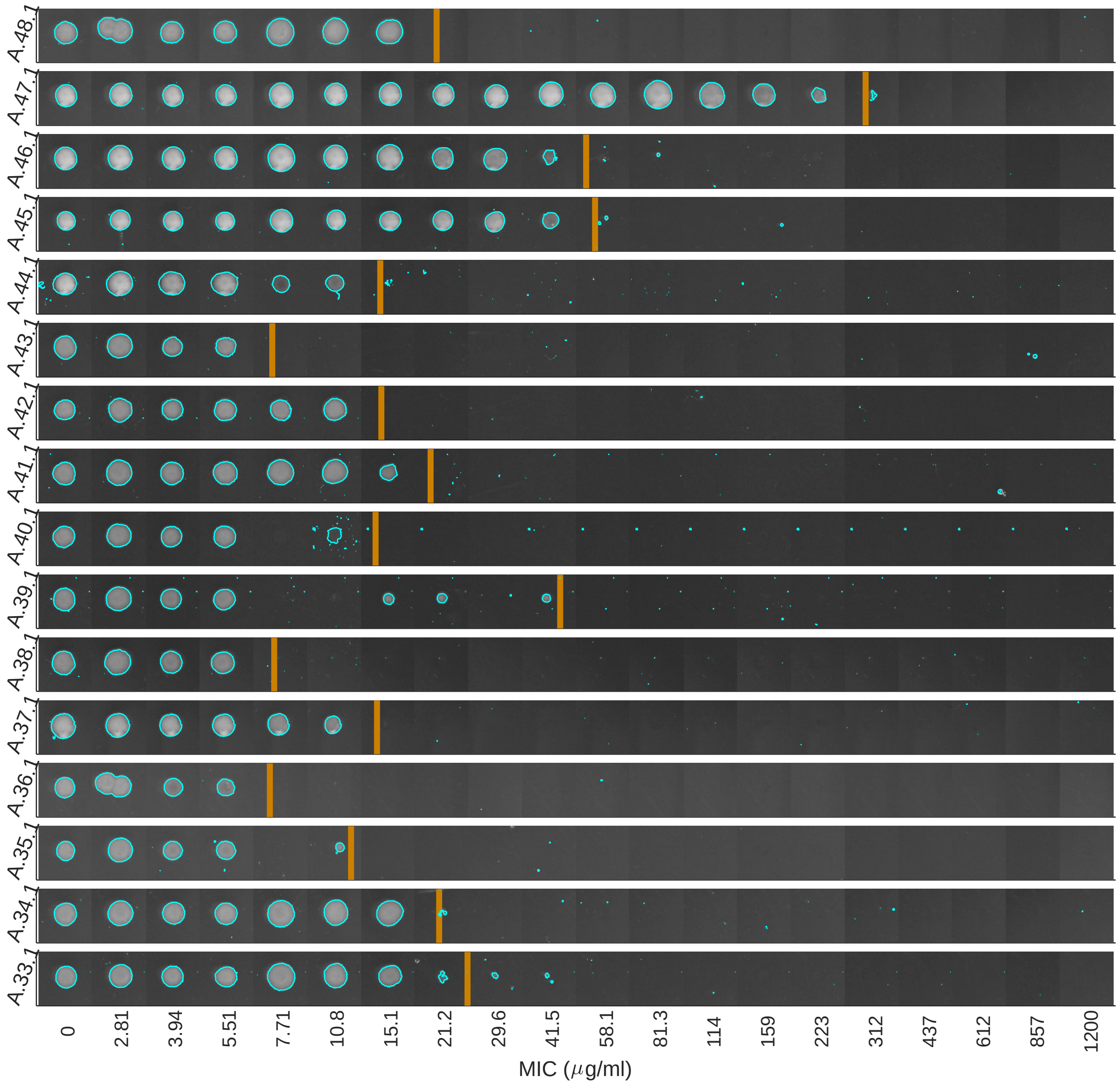

Supplementary Datasets 1: Ampicillin resistance measurements for isolates collected in Experiment A. 9/36

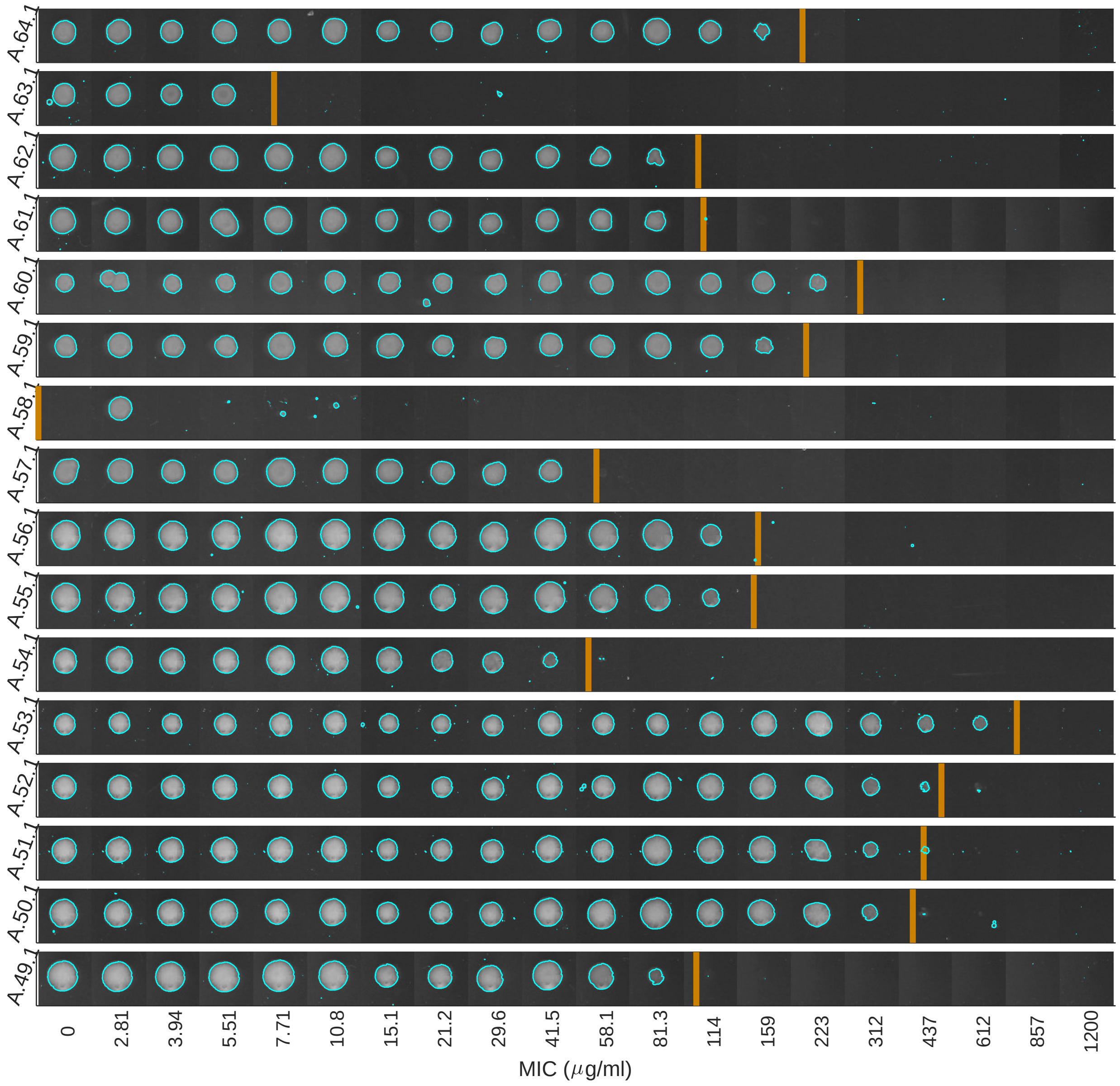

Supplementary Datasets 1: Ampicillin resistance measurements for isolates collected in Experiment A. 10/36

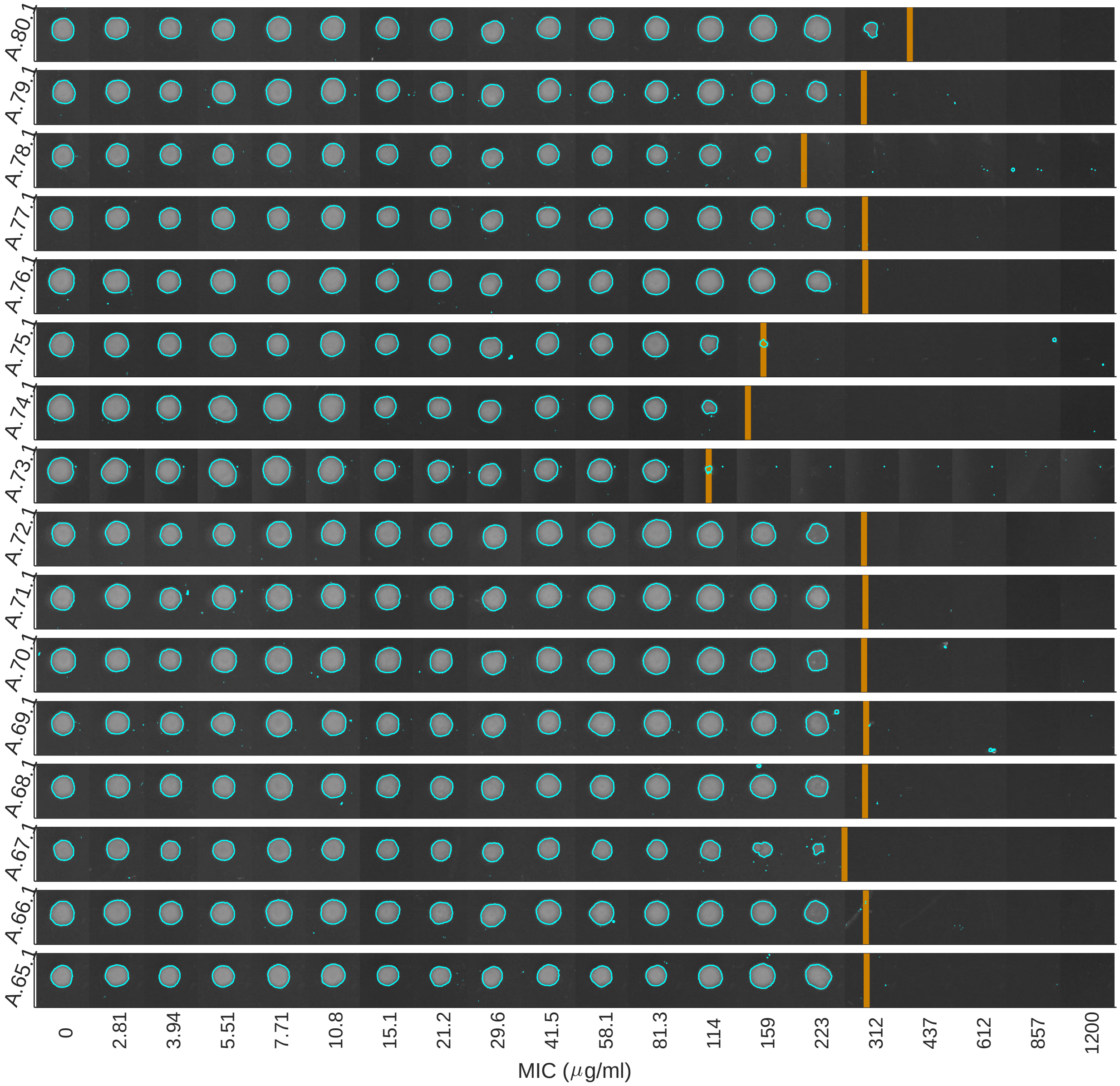

Supplementary Datasets 1: Ampicillin resistance measurements for isolates collected in Experiment A.

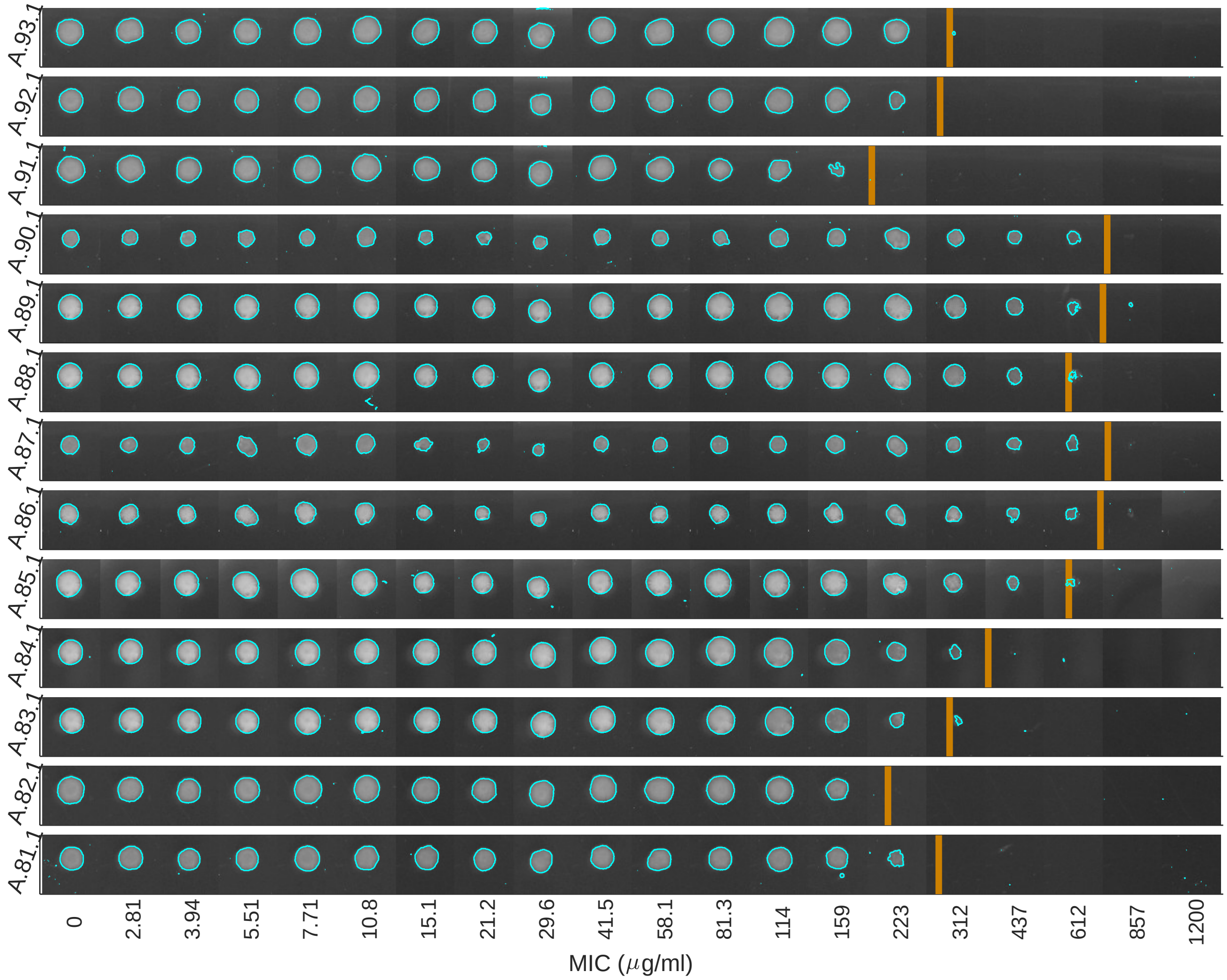

Supplementary Datasets 1: Ampicillin resistance measurements for isolates collected in Experiment A. 12/36

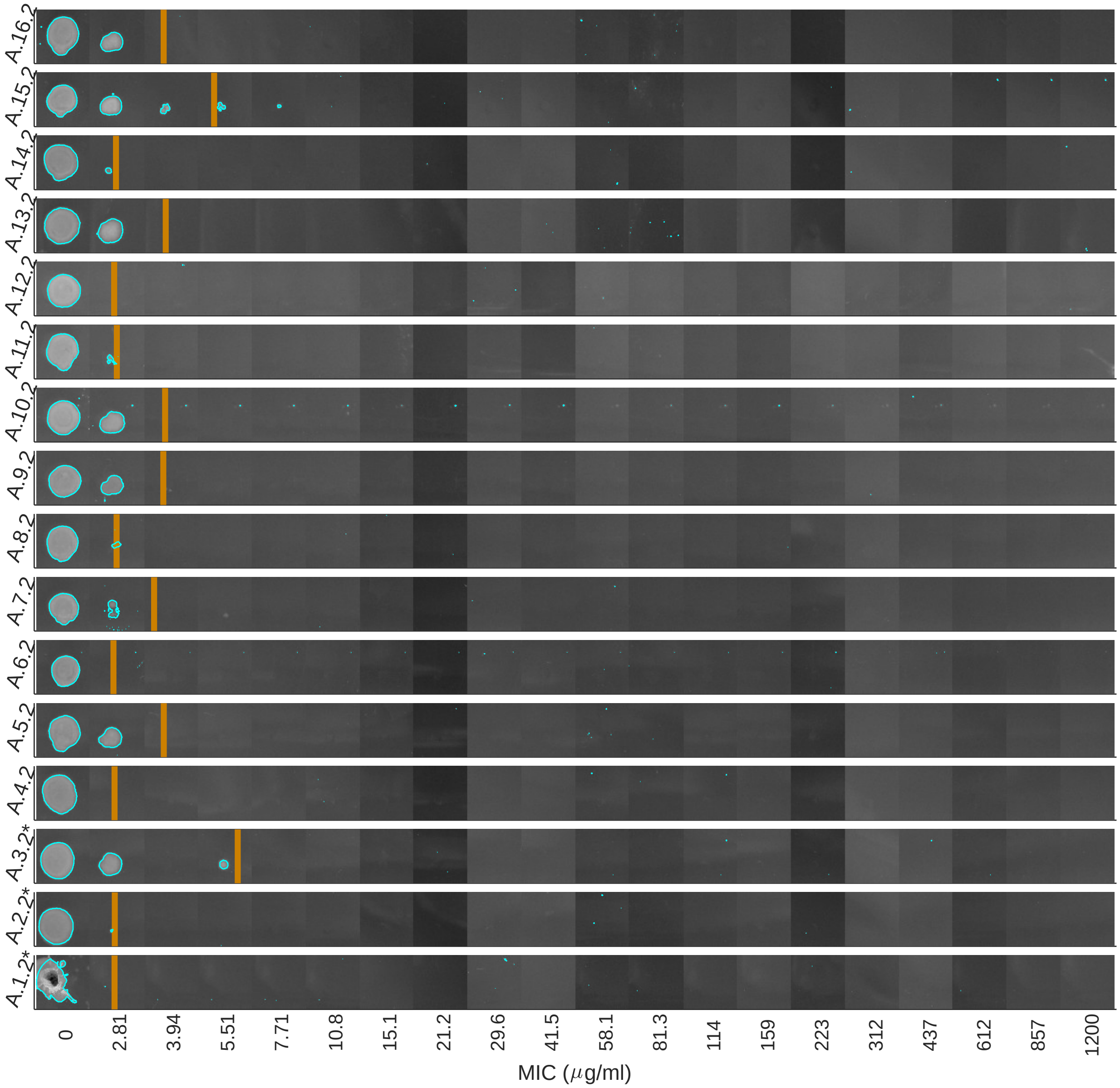

Supplementary Datasets 1: Ampicillin resistance measurements for isolates collected in Experiment A. 13/36

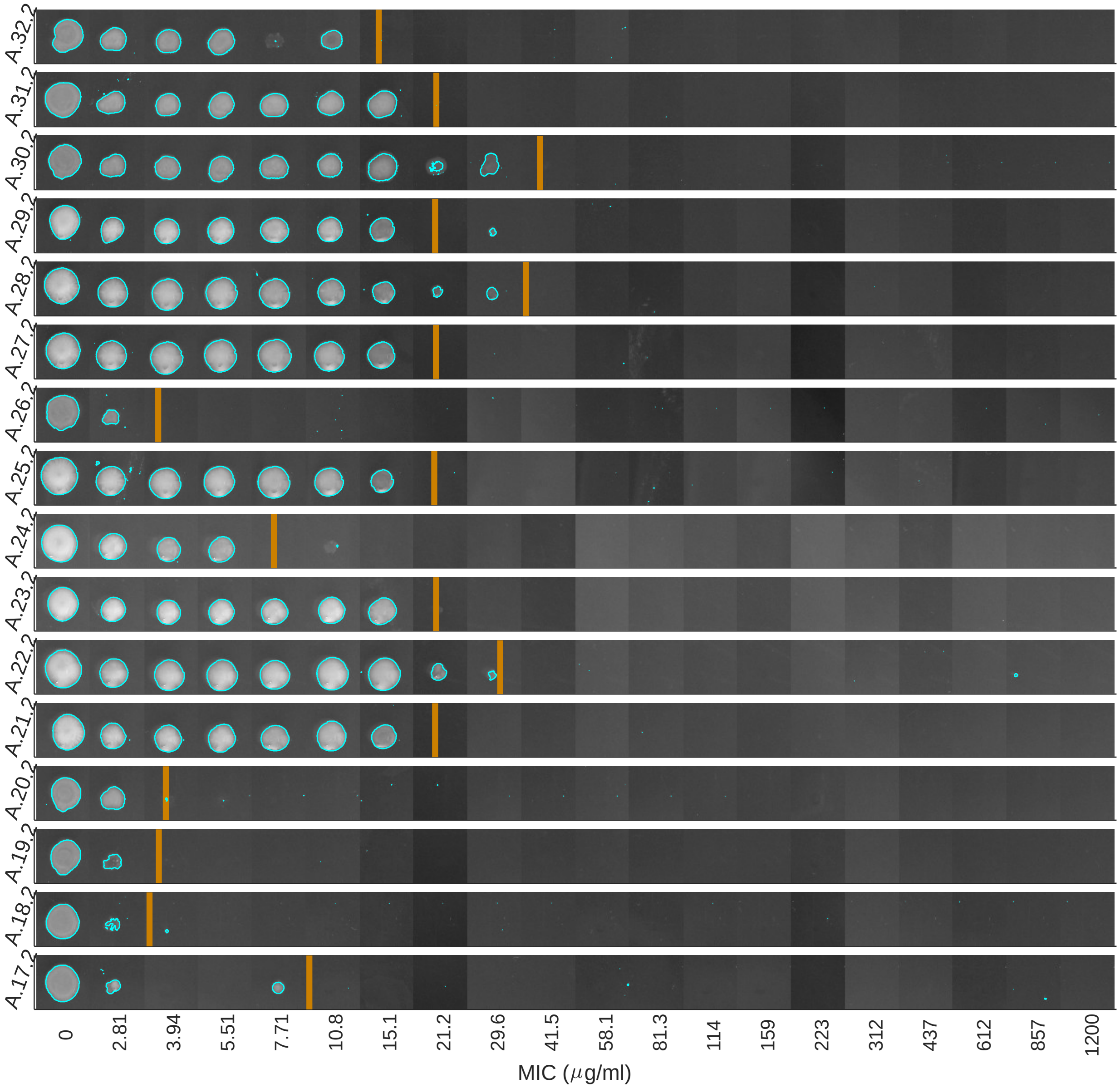

Supplementary Datasets 1: Ampicillin resistance measurements for isolates collected in Experiment A. 14/36

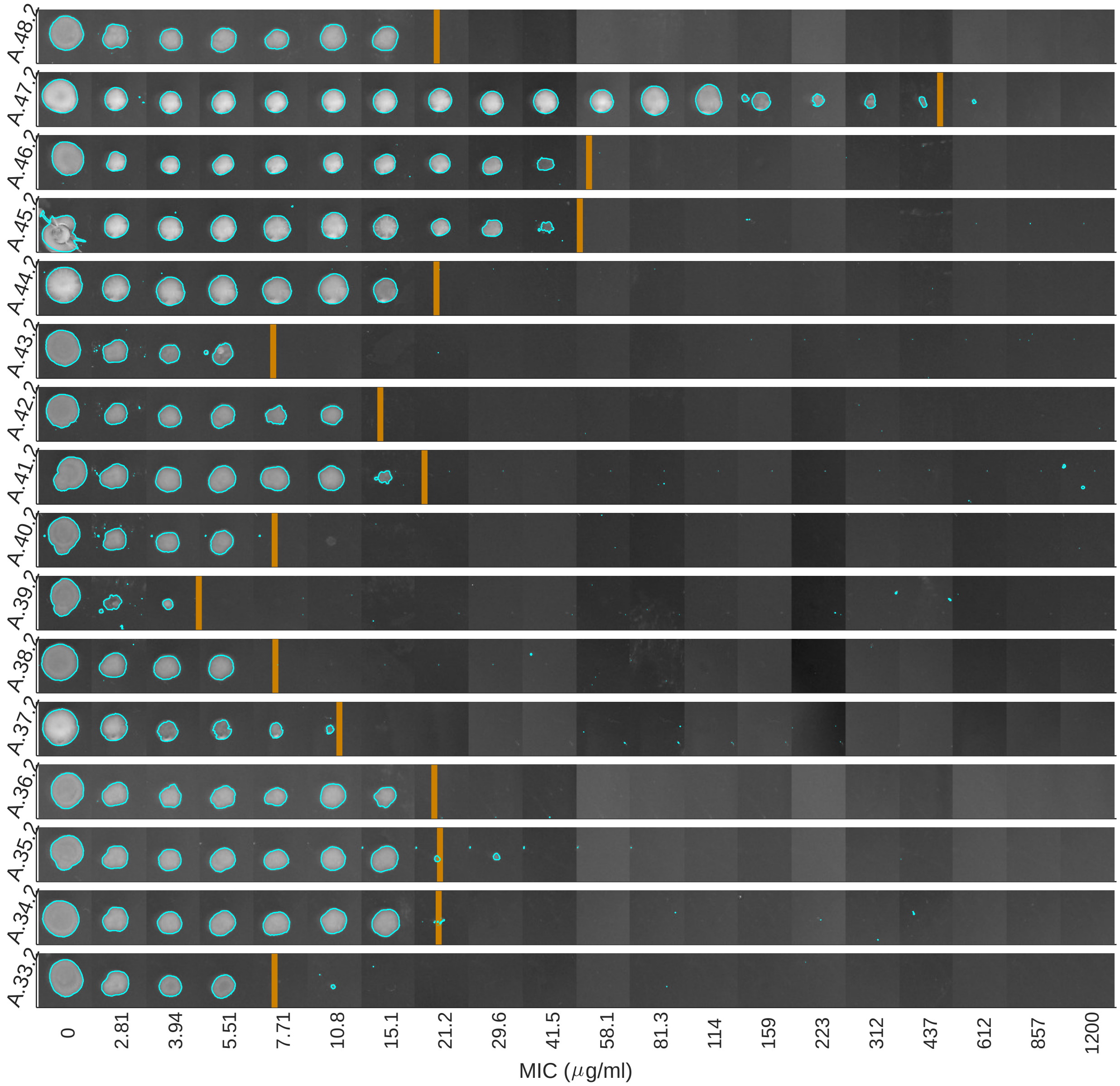

Supplementary Datasets 1: Ampicillin resistance measurements for isolates collected in Experiment A. 15/36

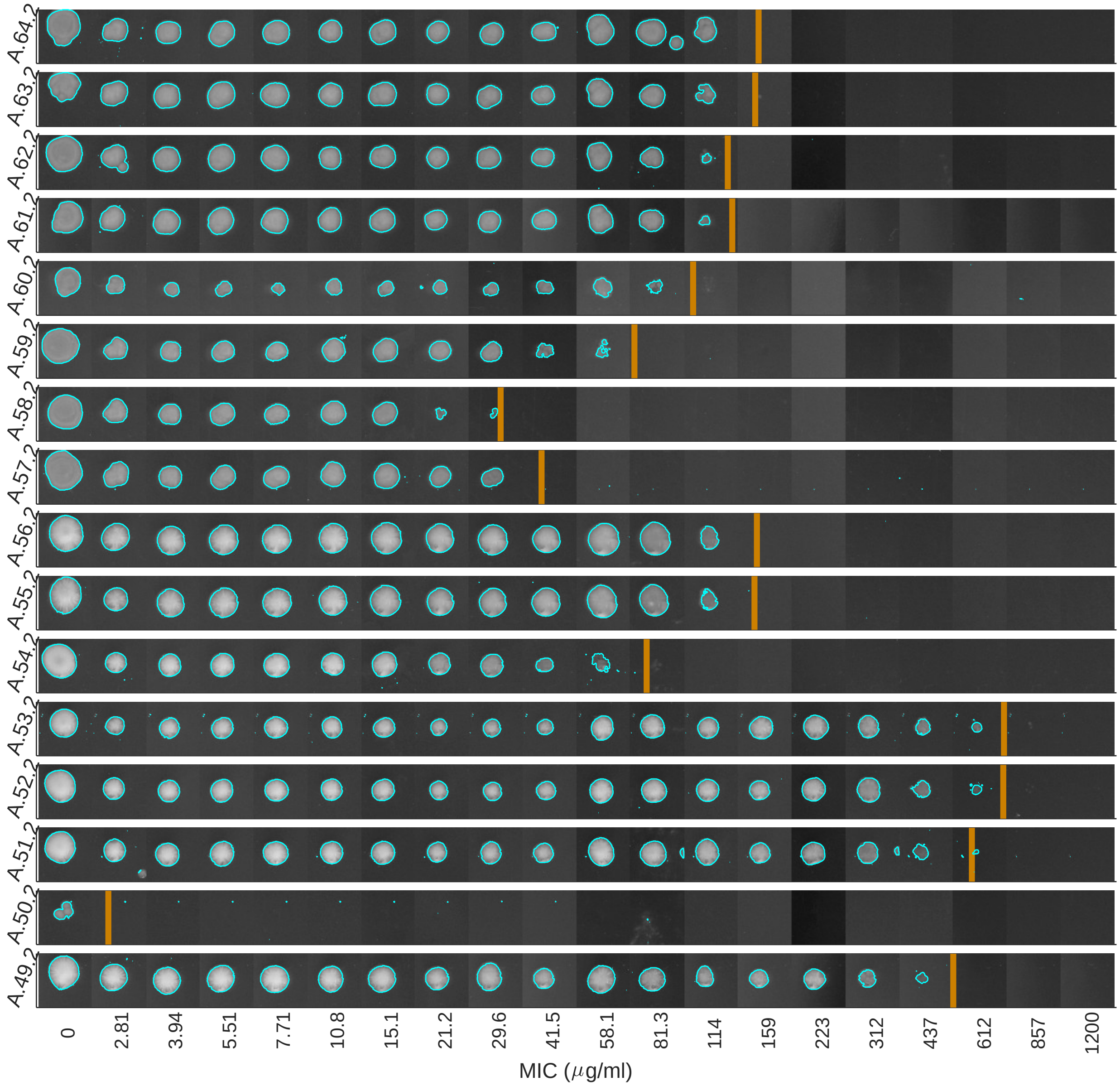

Supplementary Datasets 1: Ampicillin resistance measurements for isolates collected in Experiment A. 16/36

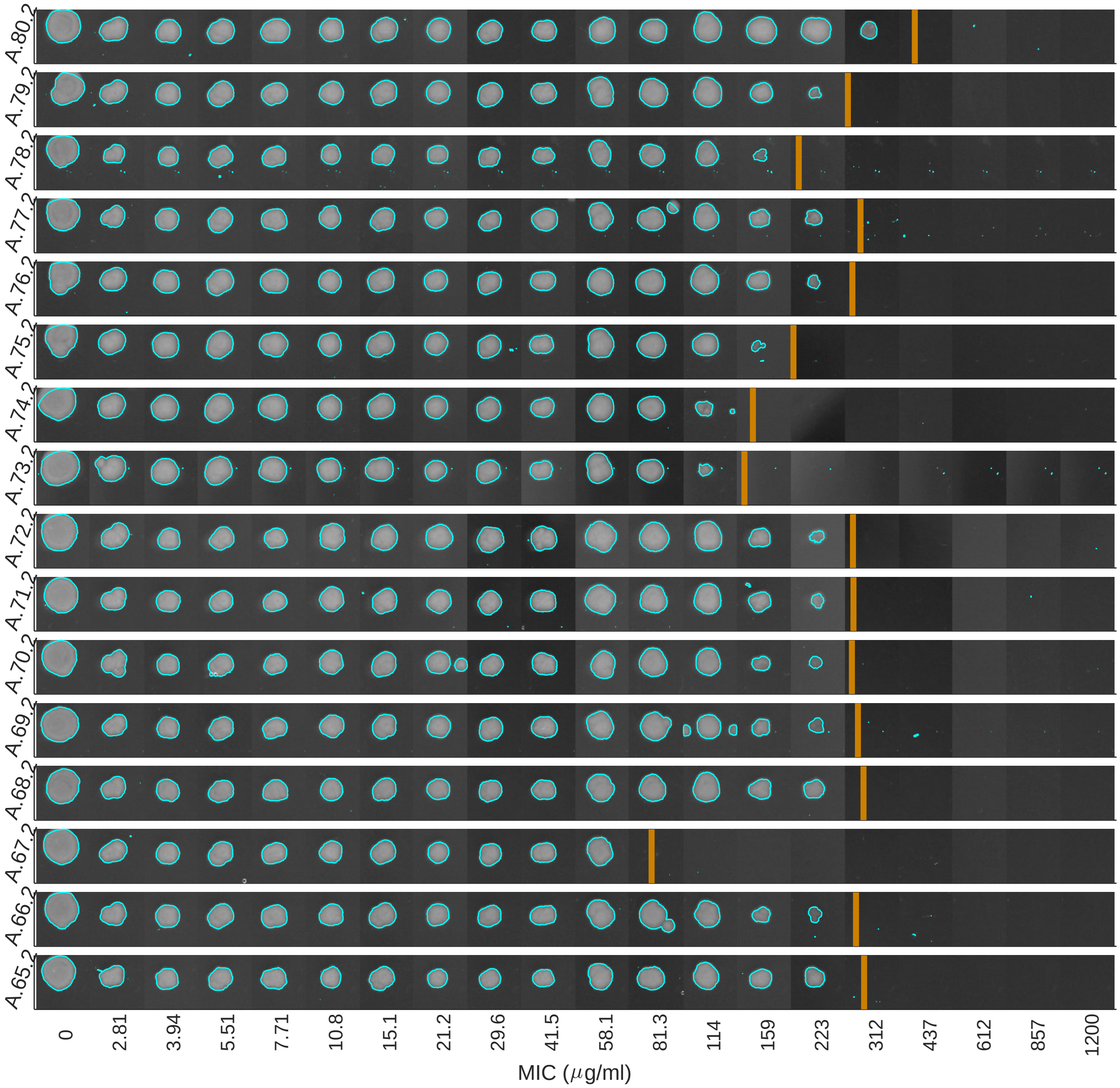

Supplementary Datasets 1: Ampicillin resistance measurements for isolates collected in Experiment A.

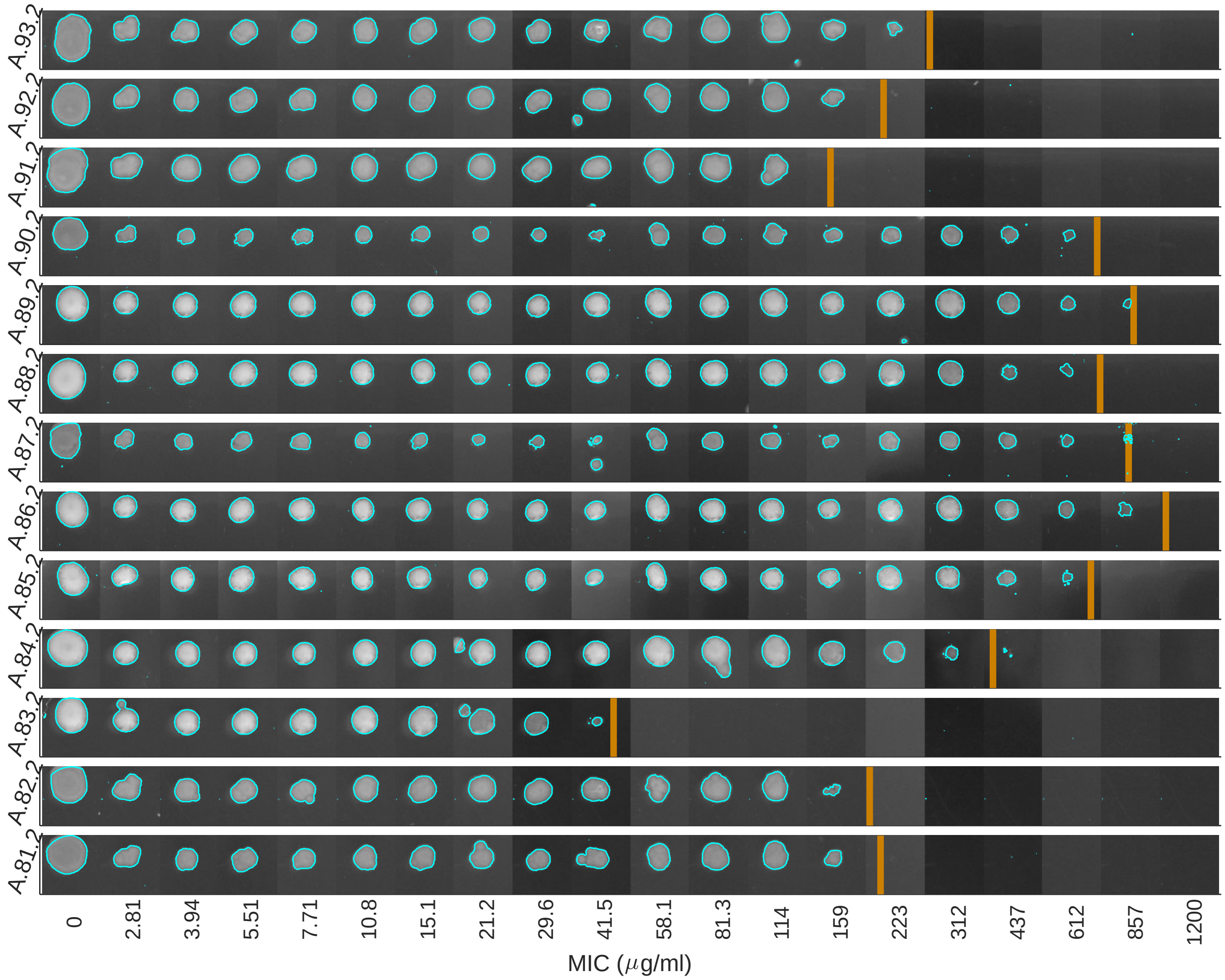

Supplementary Datasets 1: Ampicillin resistance measurements for isolates collected in Experiment A.

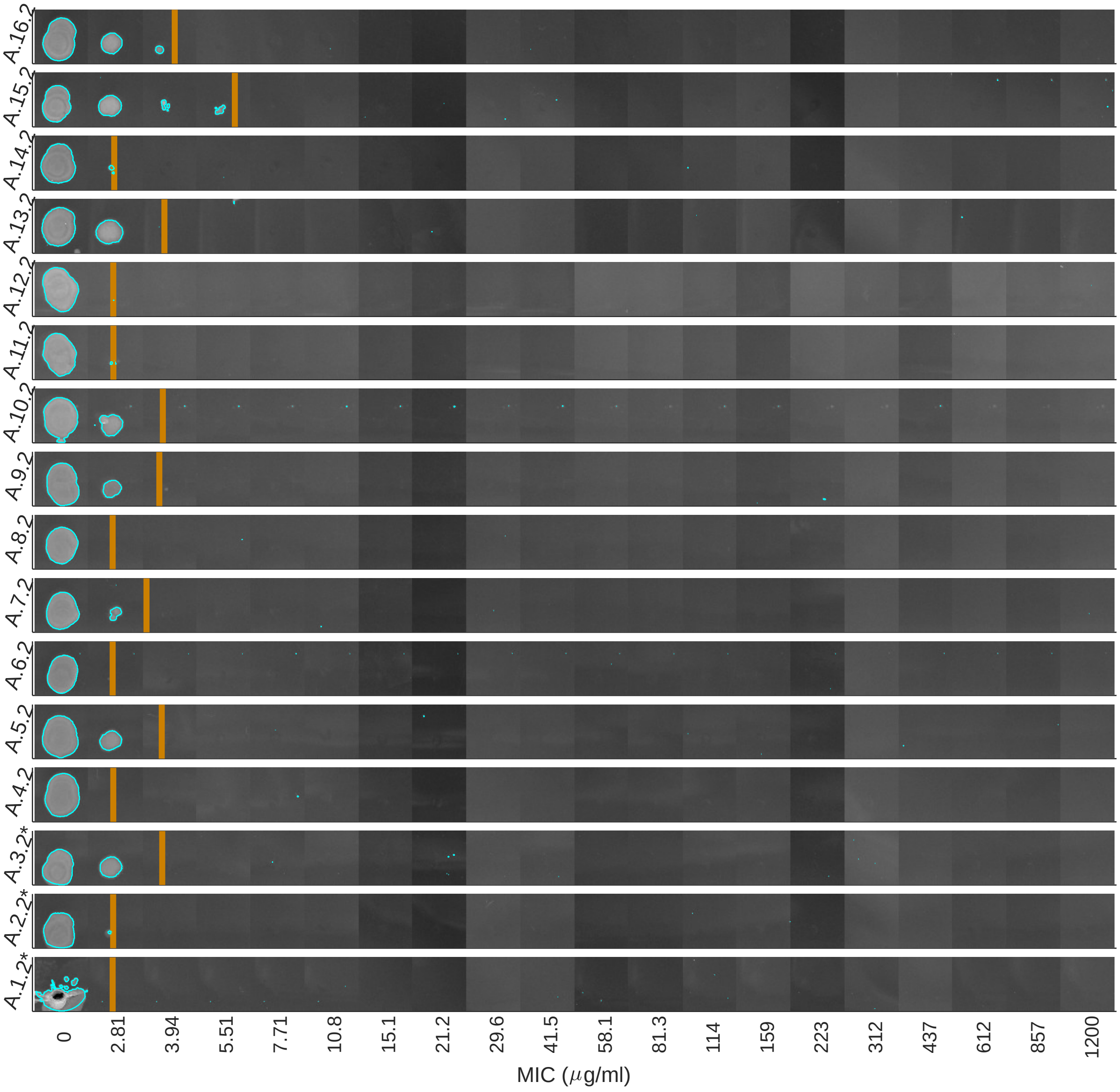

Supplementary Datasets 1: Ampicillin resistance measurements for isolates collected in Experiment A.

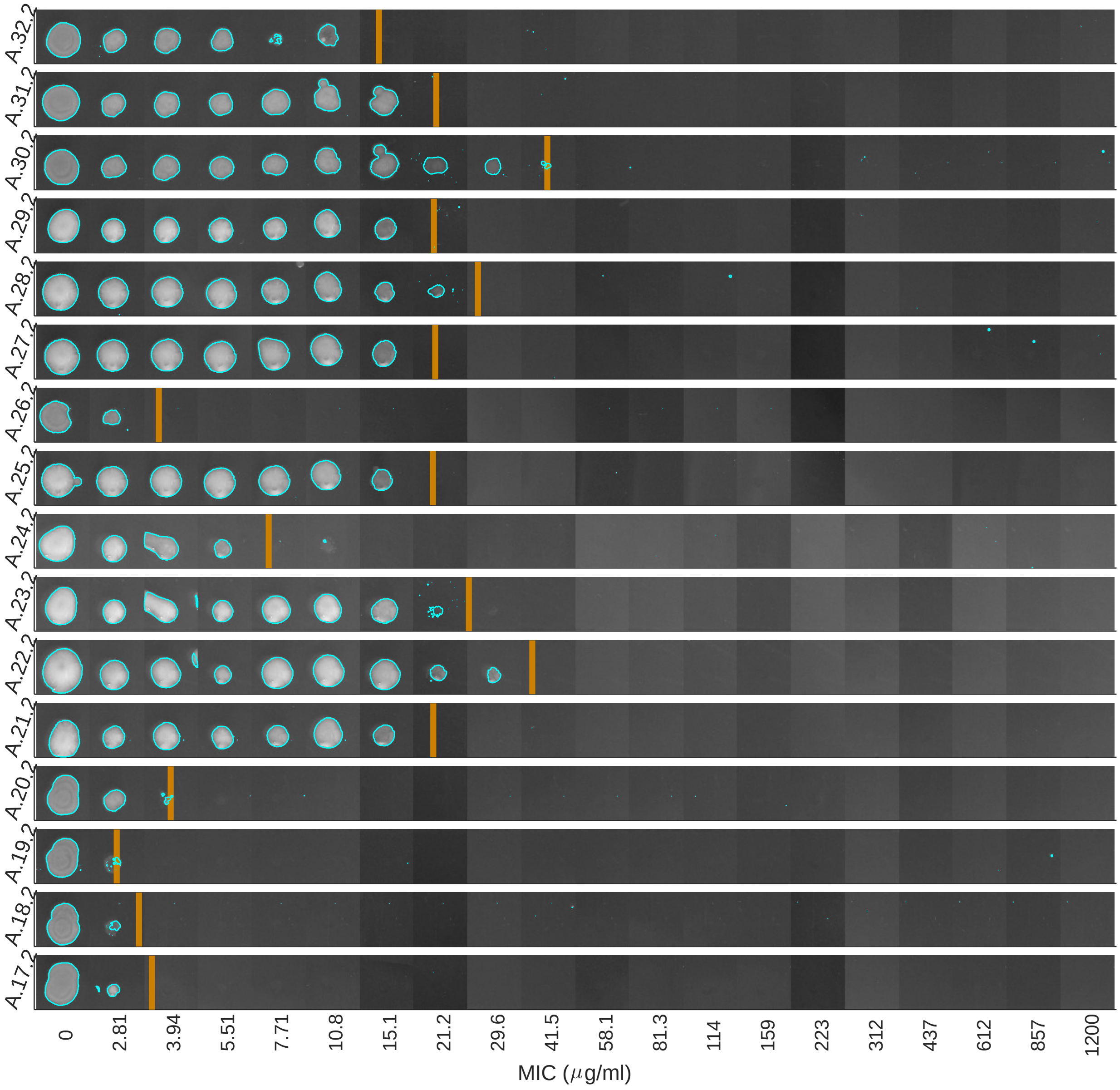

Supplementary Datasets 1: Ampicillin resistance measurements for isolates collected in Experiment A. 20/36

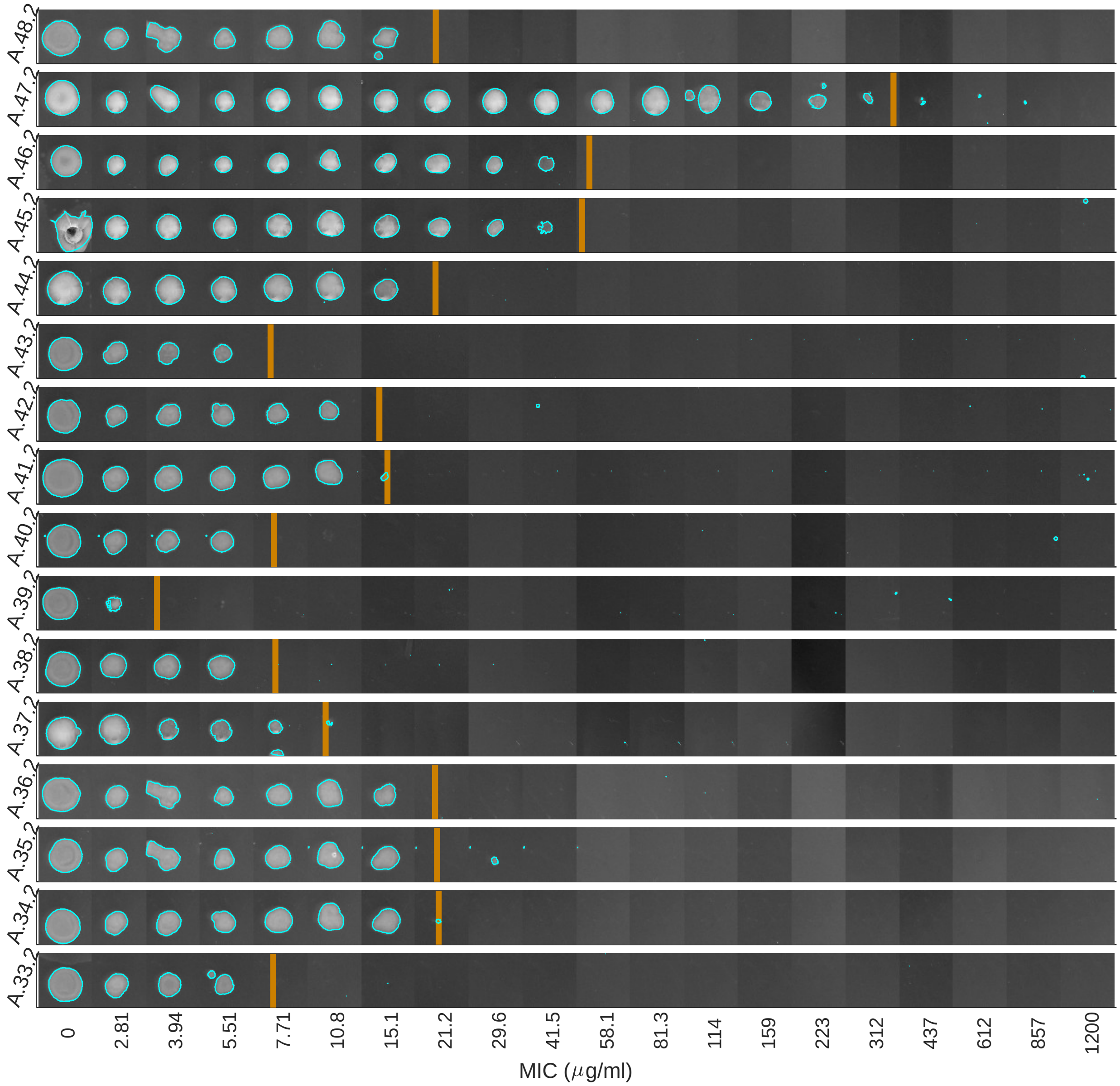

Supplementary Datasets 1: Ampicillin resistance measurements for isolates collected in Experiment A. 21/36

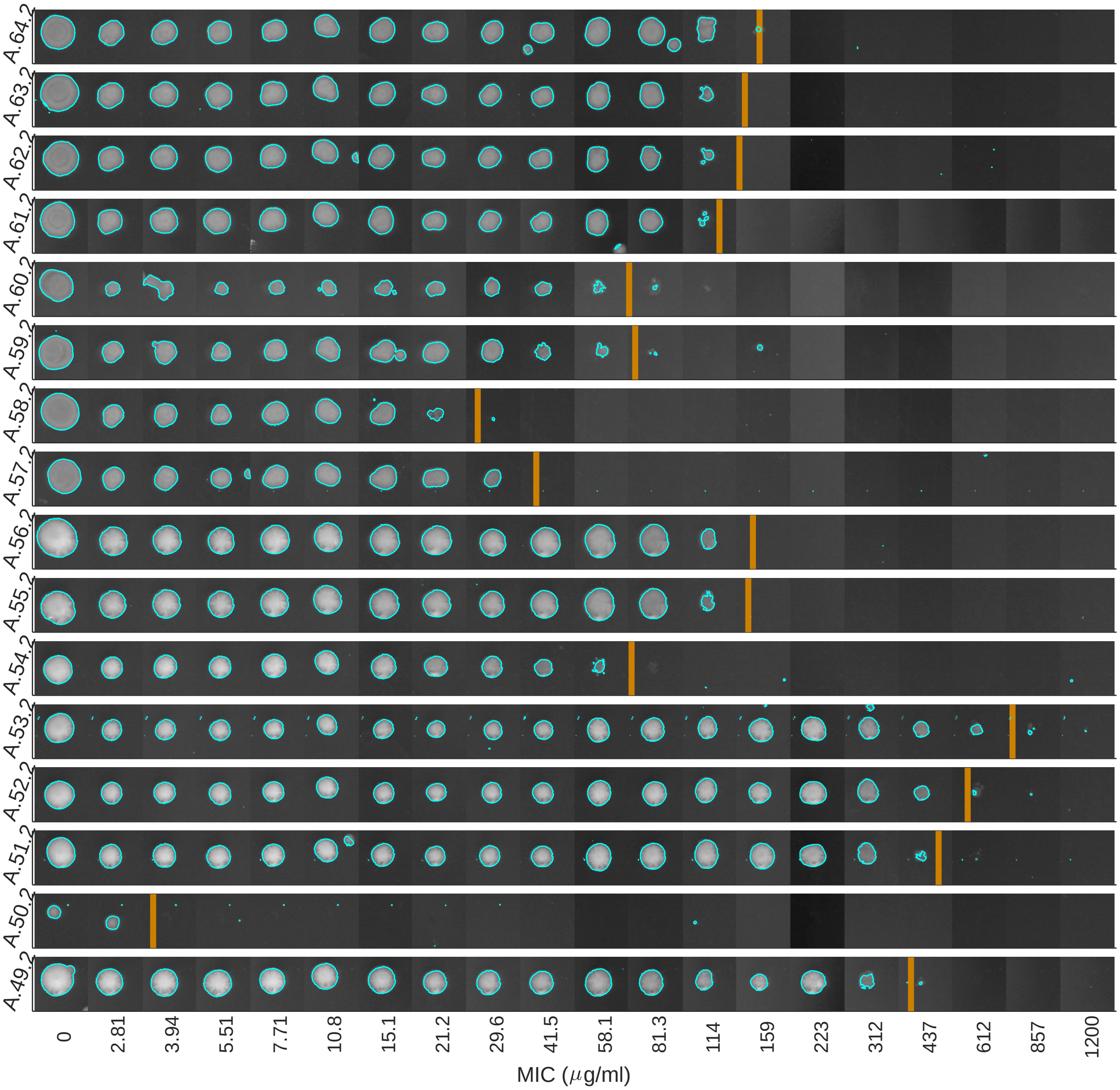

Supplementary Datasets 1: Ampicillin resistance measurements for isolates collected in Experiment A. 22/36

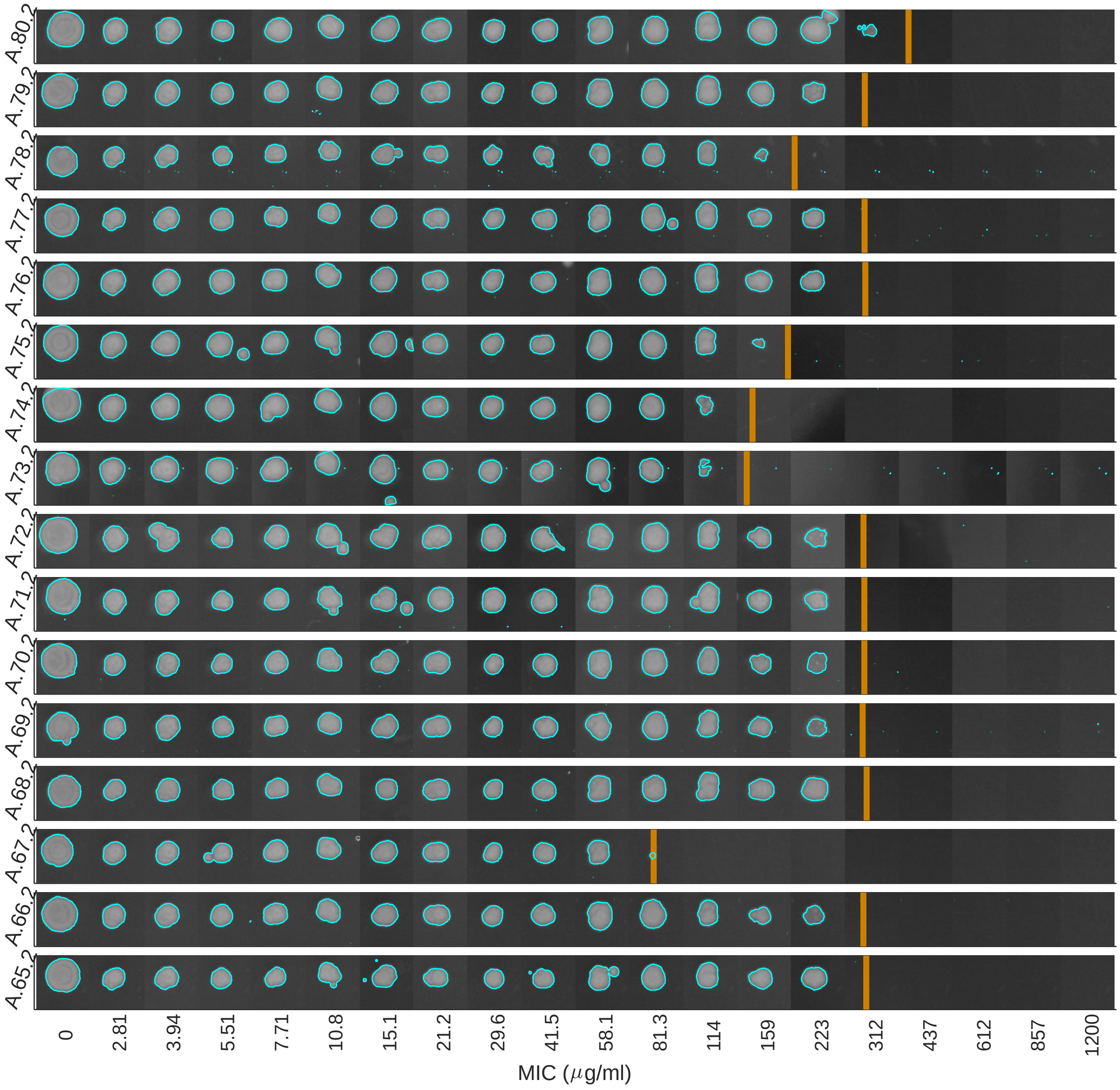

Supplementary Datasets 1: Ampicillin resistance measurements for isolates collected in Experiment A. 23/36

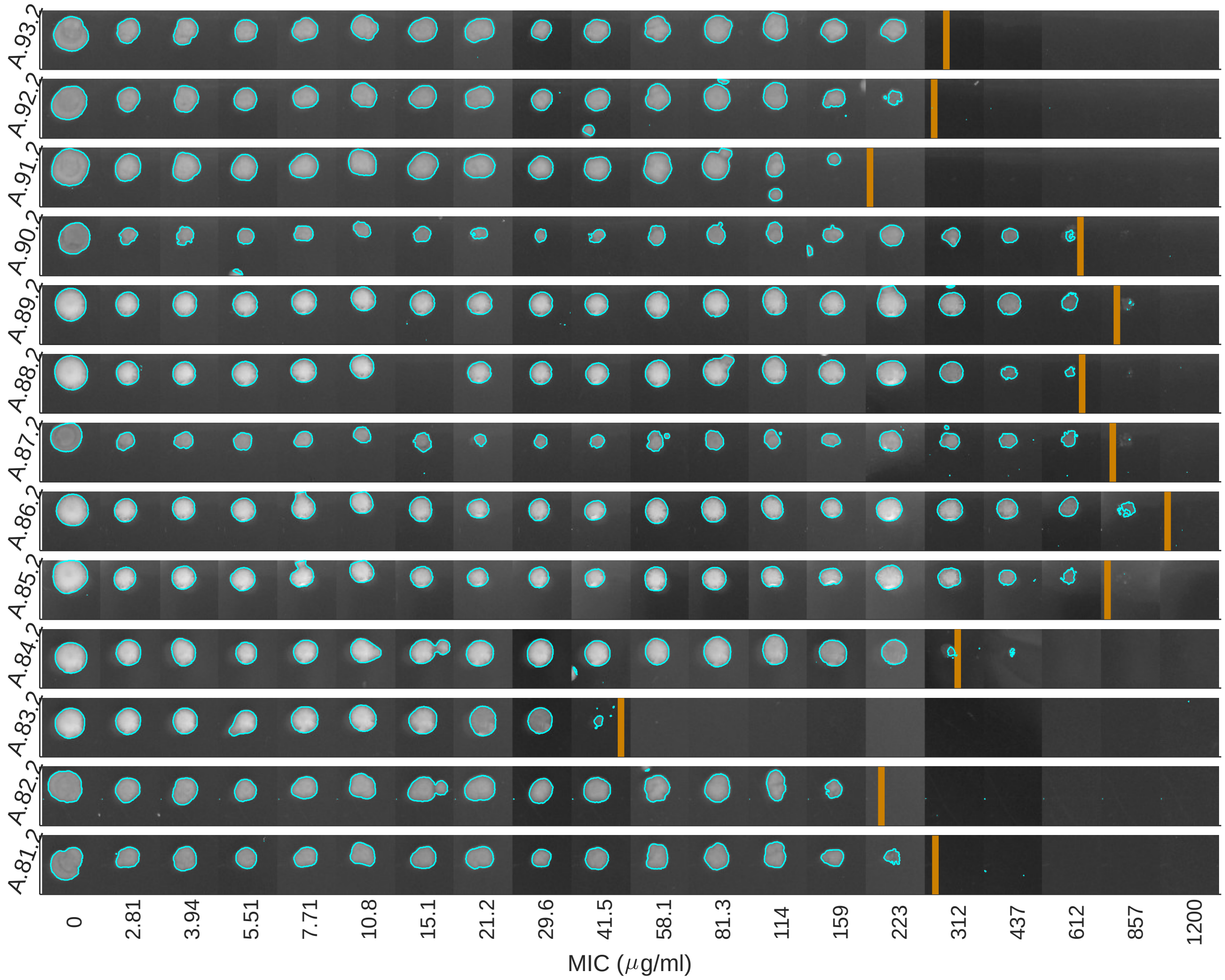

Supplementary Datasets 1: Ampicillin resistance measurements for isolates collected in Experiment A.

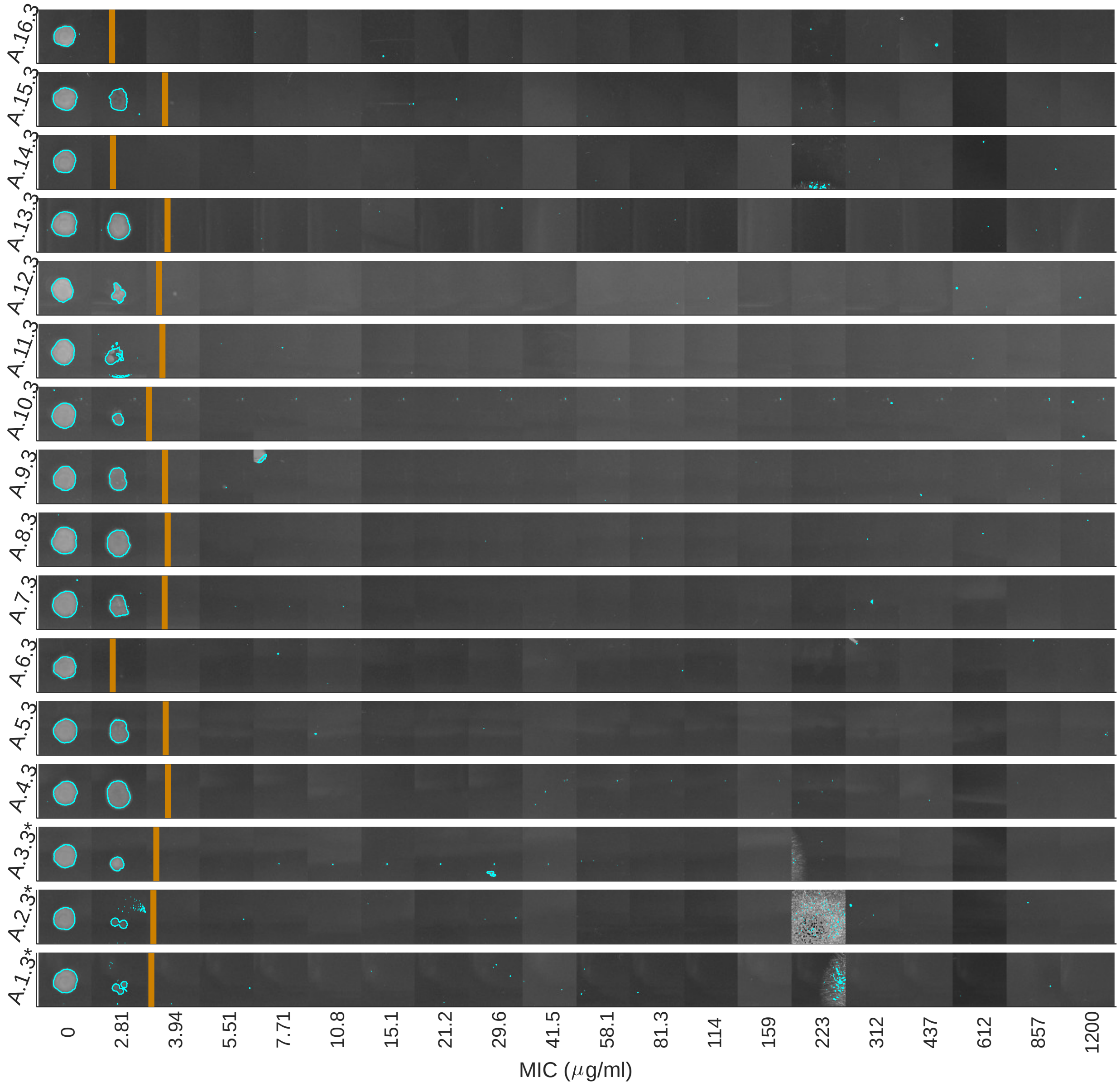

Supplementary Datasets 1: Ampicillin resistance measurements for isolates collected in Experiment A.

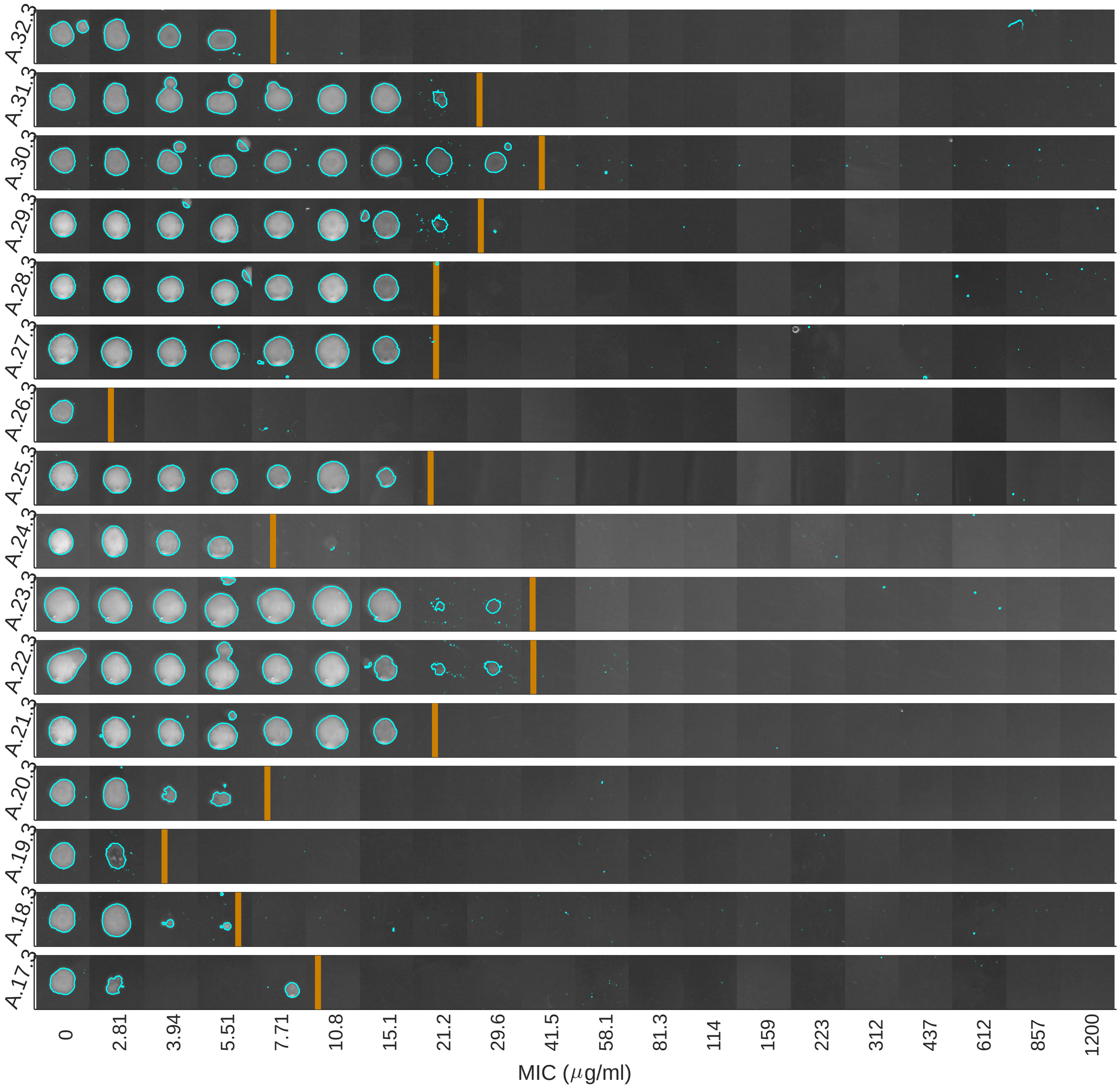

Supplementary Datasets 1: Ampicillin resistance measurements for isolates collected in Experiment A. 26/36

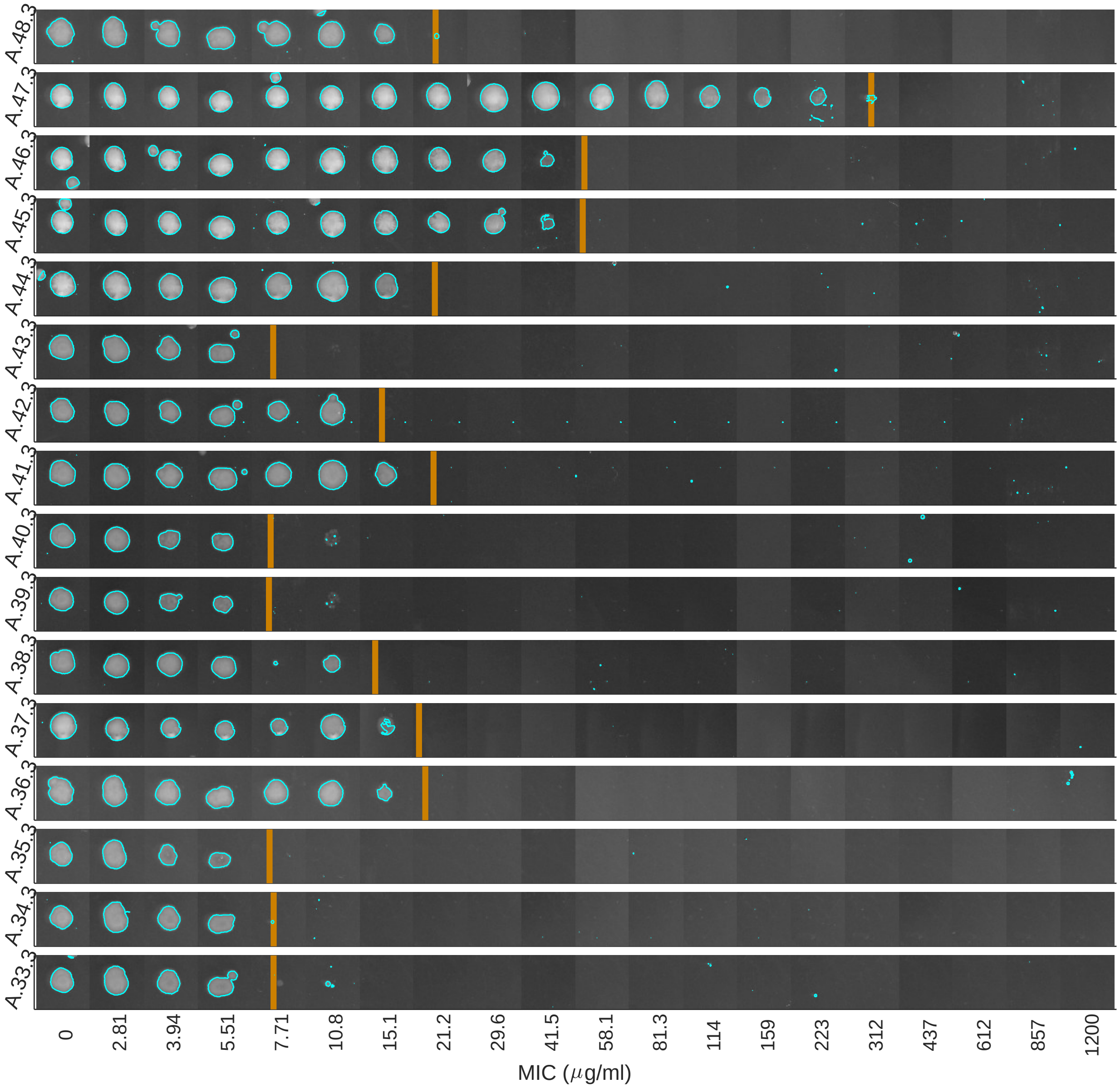

Supplementary Datasets 1: Ampicillin resistance measurements for isolates collected in Experiment A.

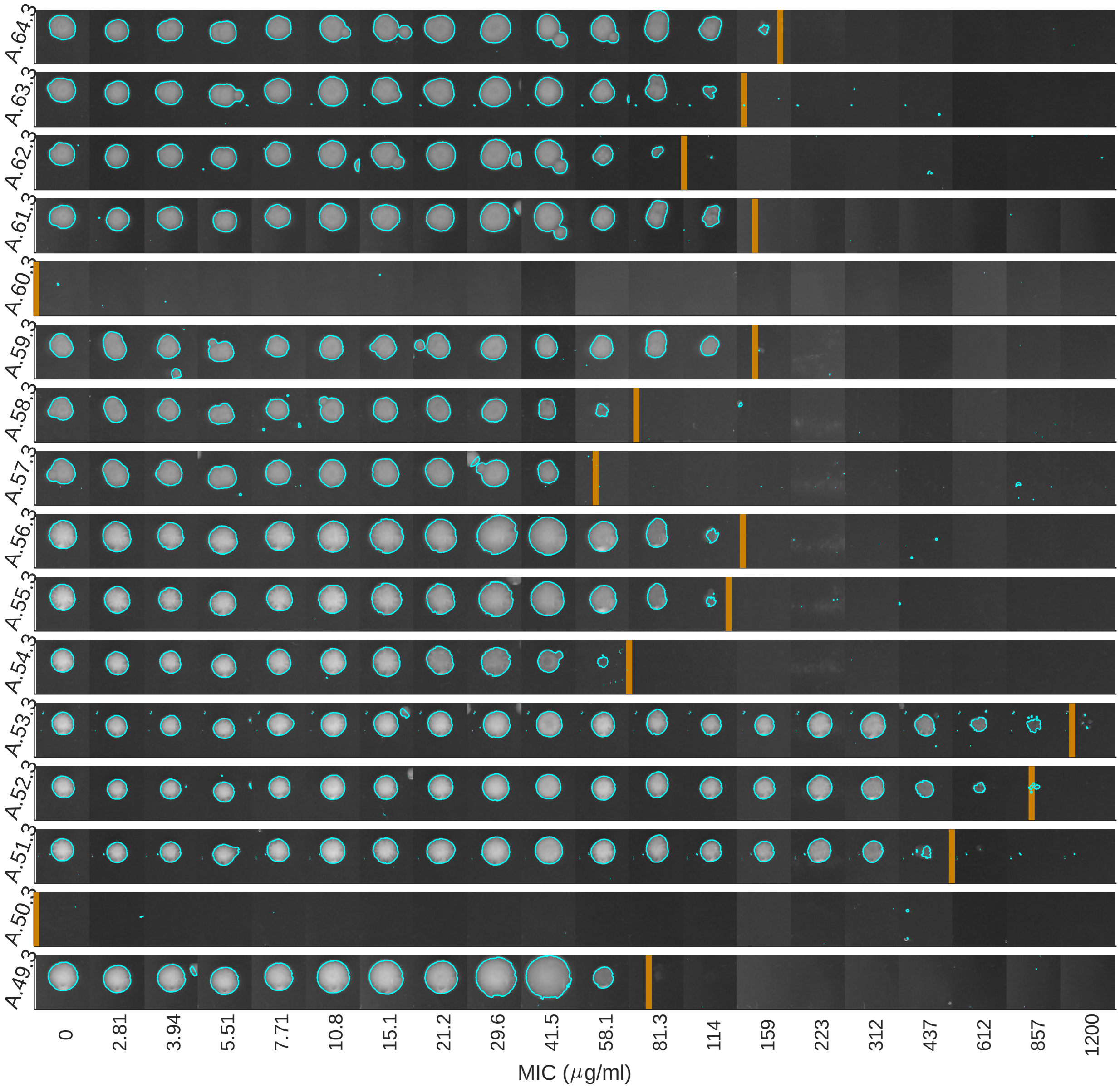

Supplementary Datasets 1: Ampicillin resistance measurements for isolates collected in Experiment A. 28/36

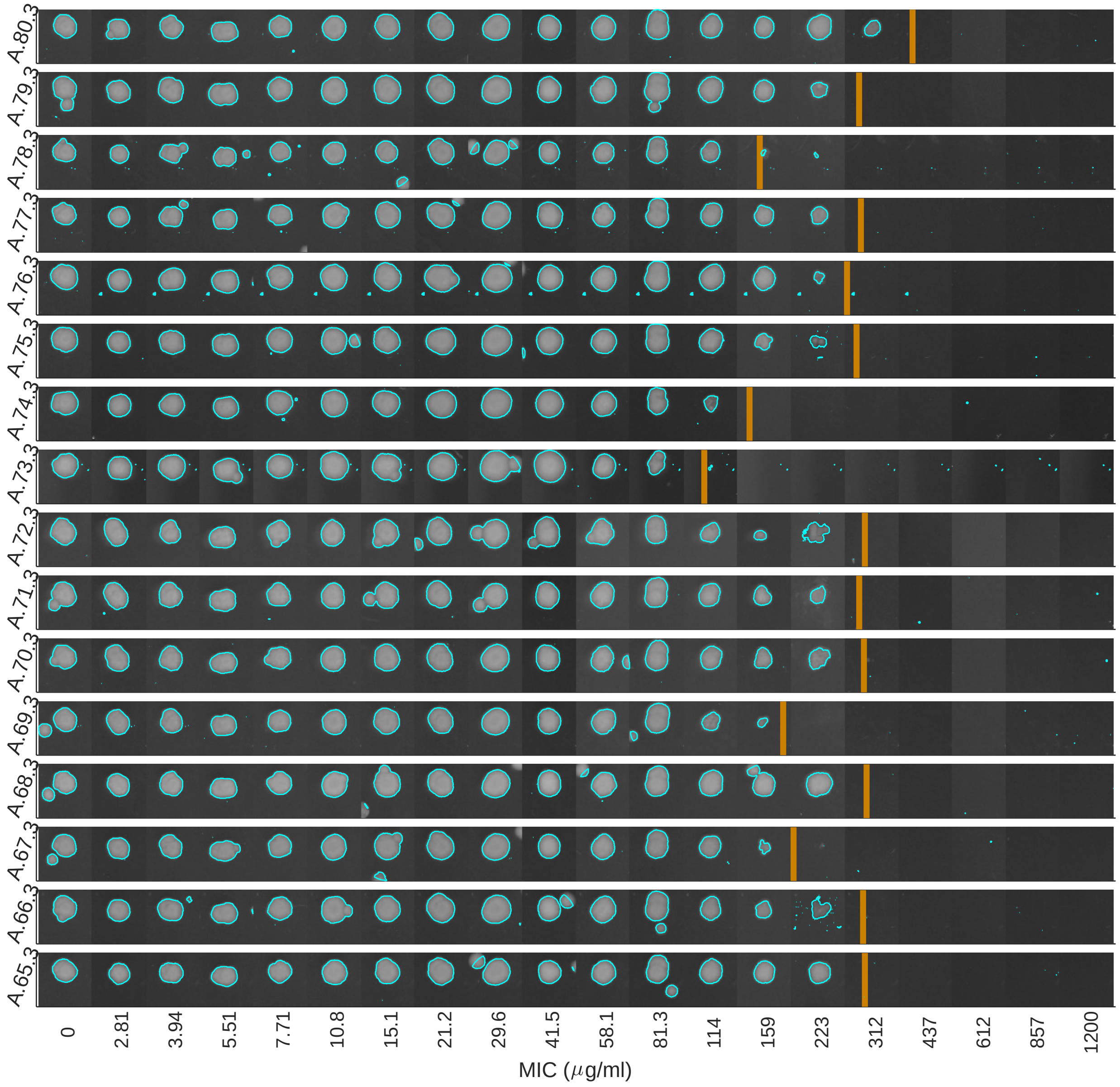

Supplementary Datasets 1: Ampicillin resistance measurements for isolates collected in Experiment A.

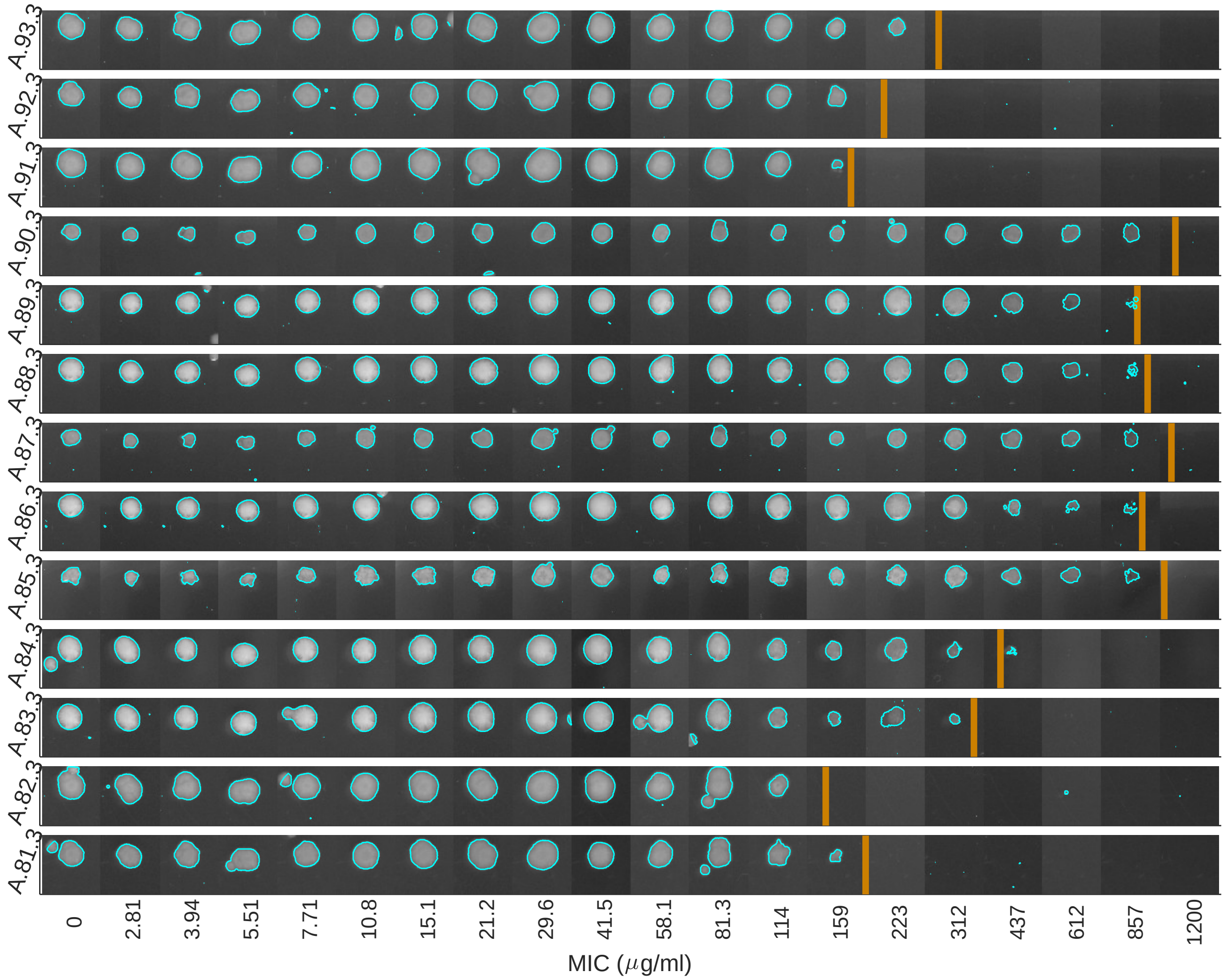

Supplementary Datasets 1: Ampicillin resistance measurements for isolates collected in Experiment A. 30/36

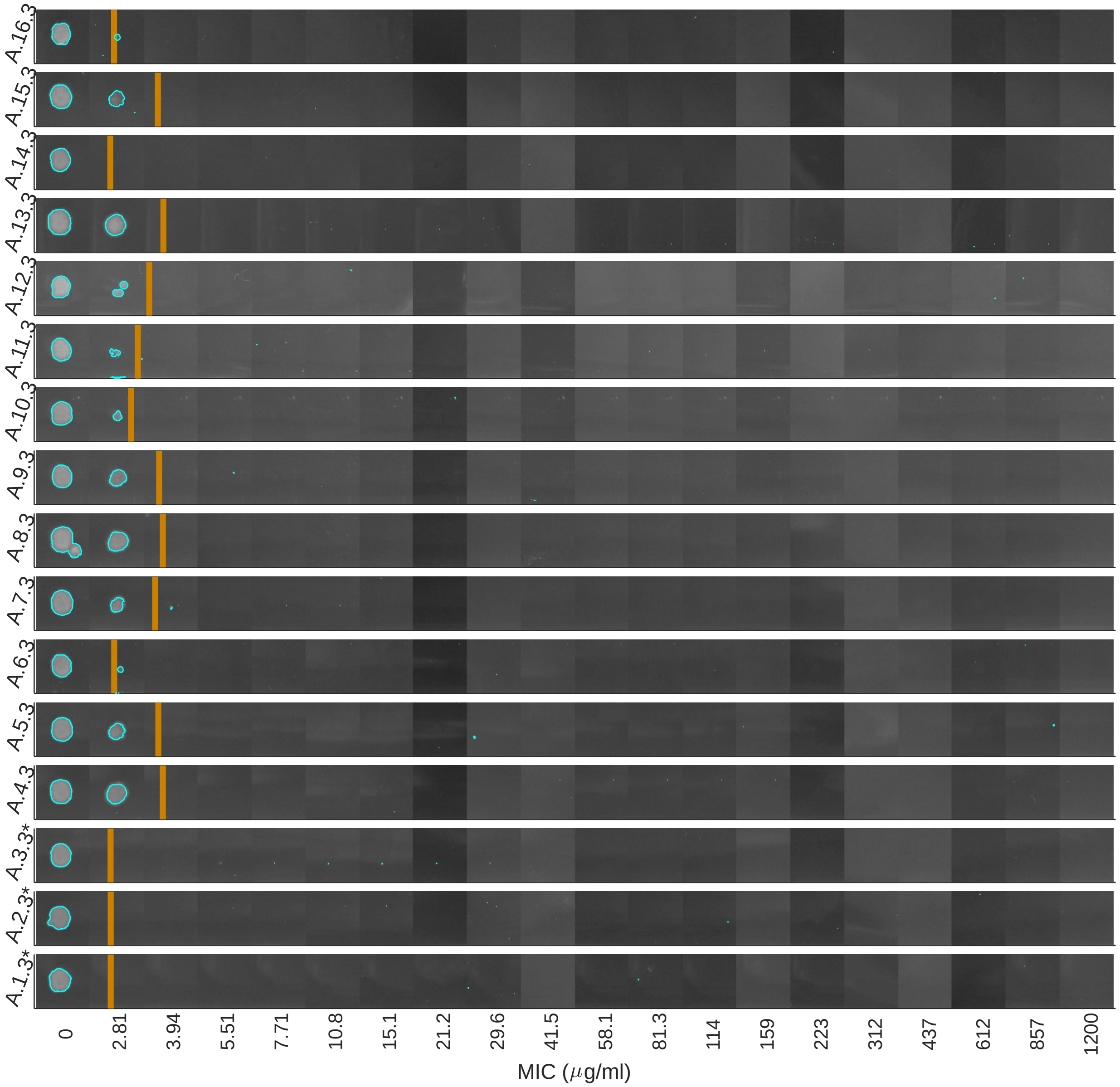

Supplementary Datasets 1: Ampicillin resistance measurements for isolates collected in Experiment A. 31/36

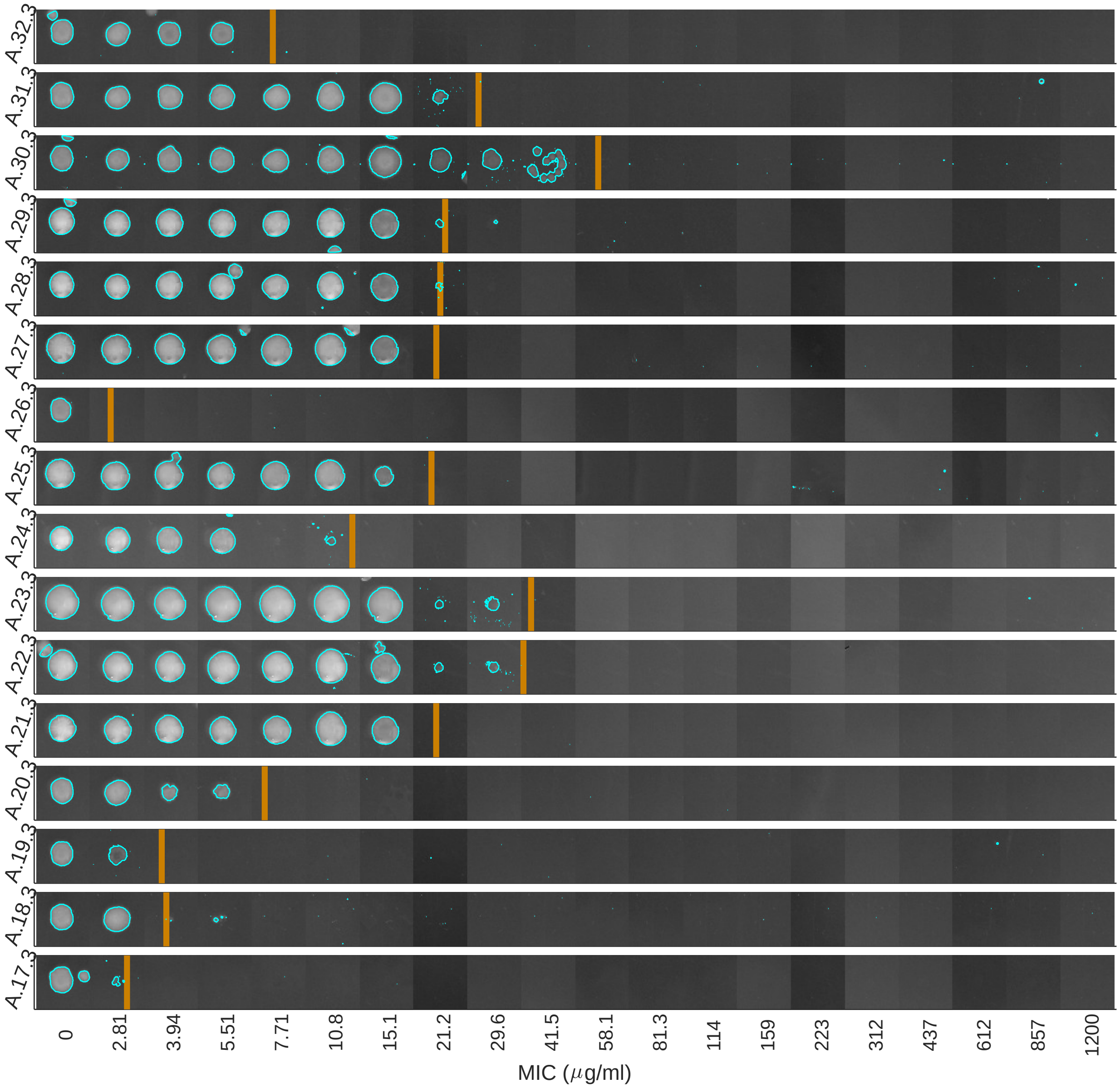

Supplementary Datasets 1: Ampicillin resistance measurements for isolates collected in Experiment A. 32/36

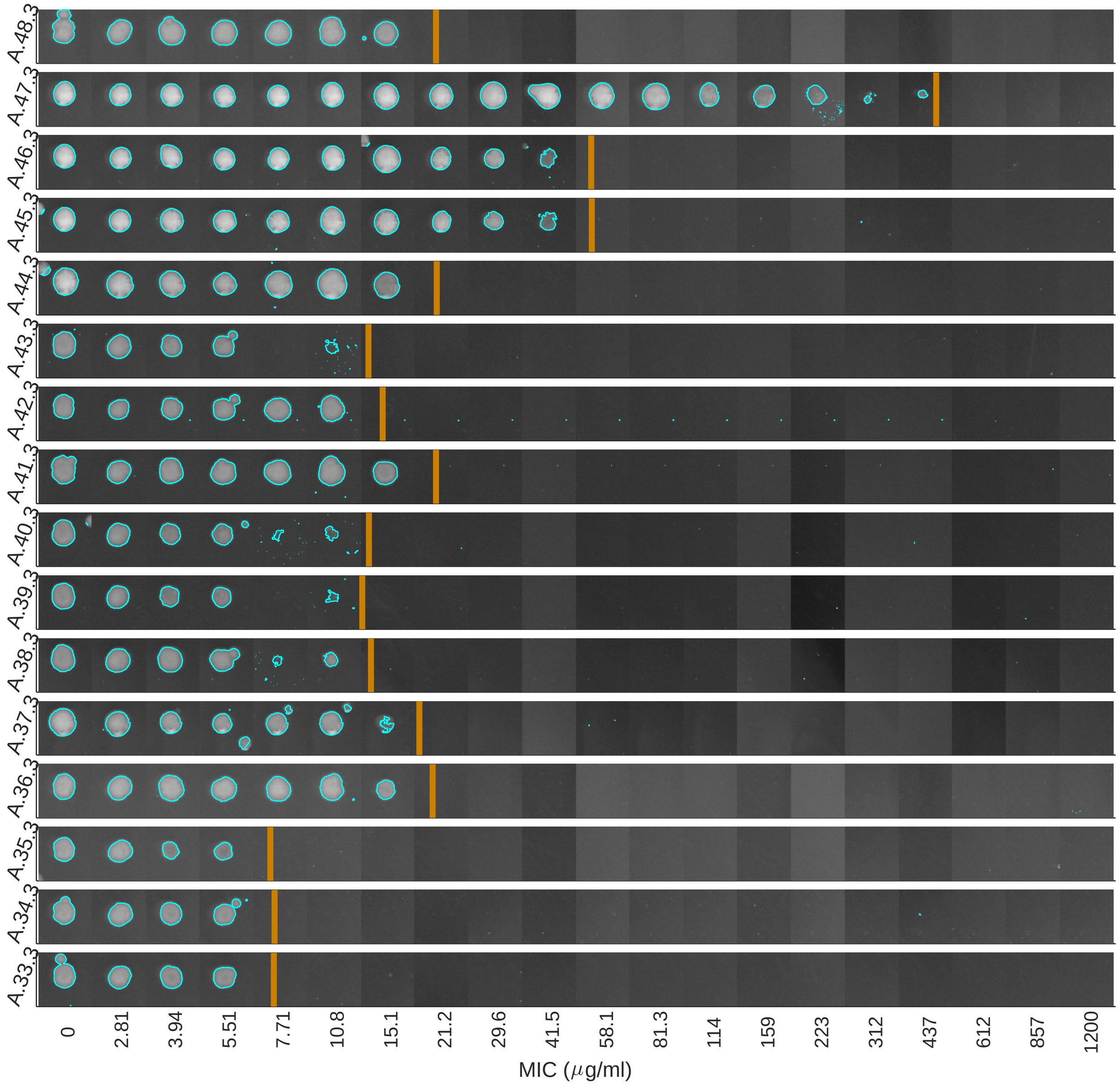

Supplementary Datasets 1: Ampicillin resistance measurements for isolates collected in Experiment A.

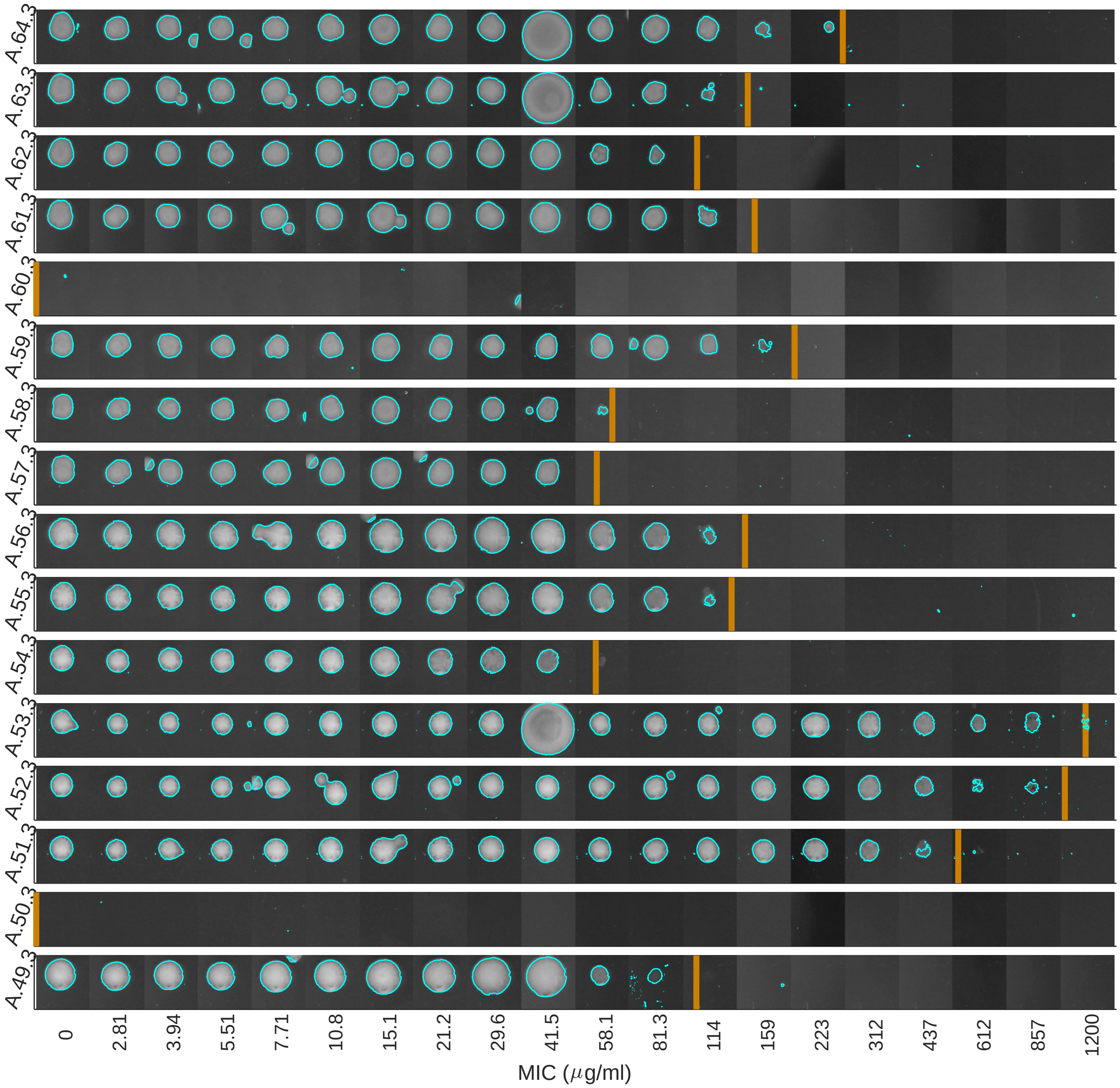

Supplementary Datasets 1: Ampicillin resistance measurements for isolates collected in Experiment A. 34/36

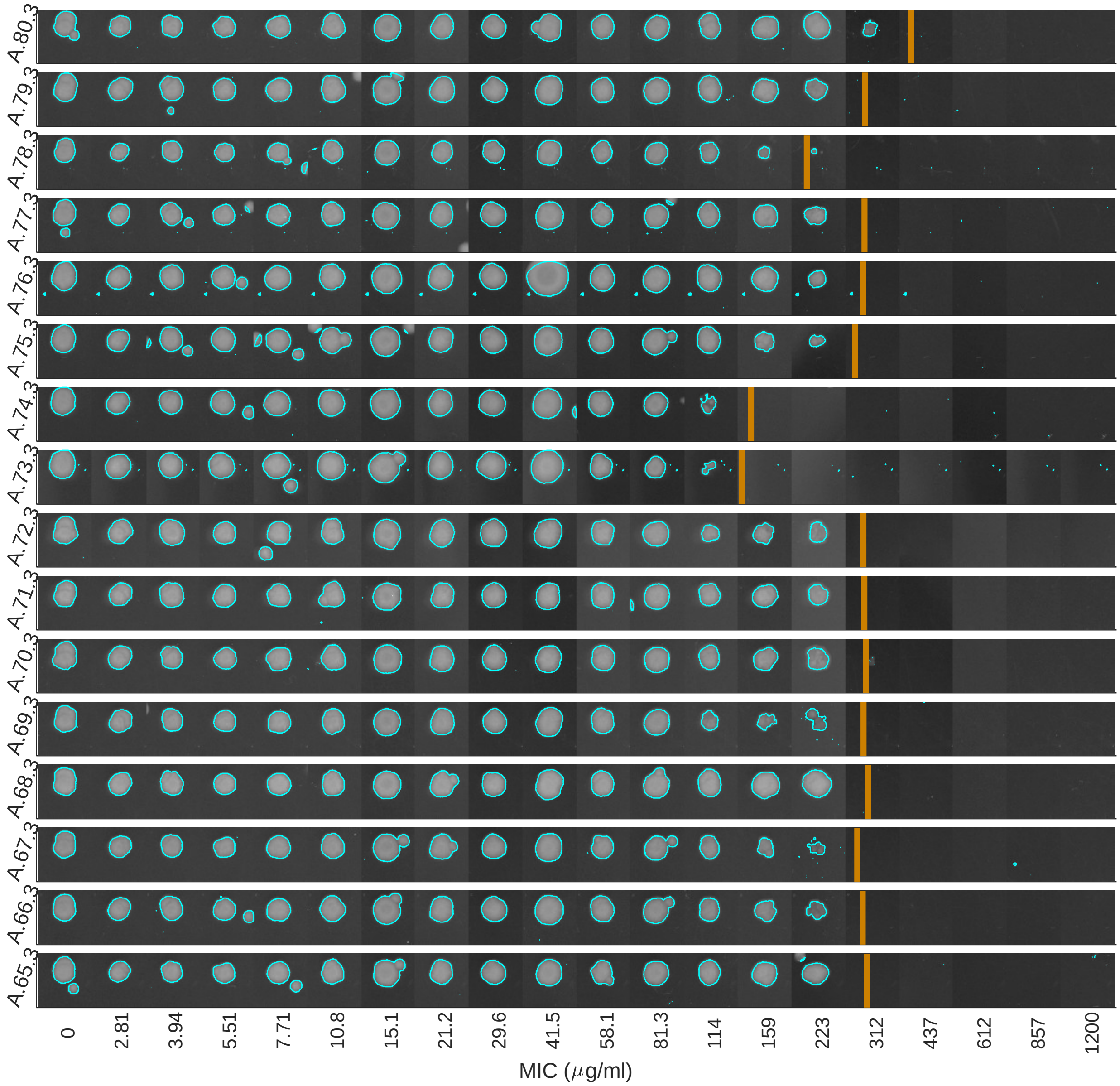

Supplementary Datasets 1: Ampicillin resistance measurements for isolates collected in Experiment A.

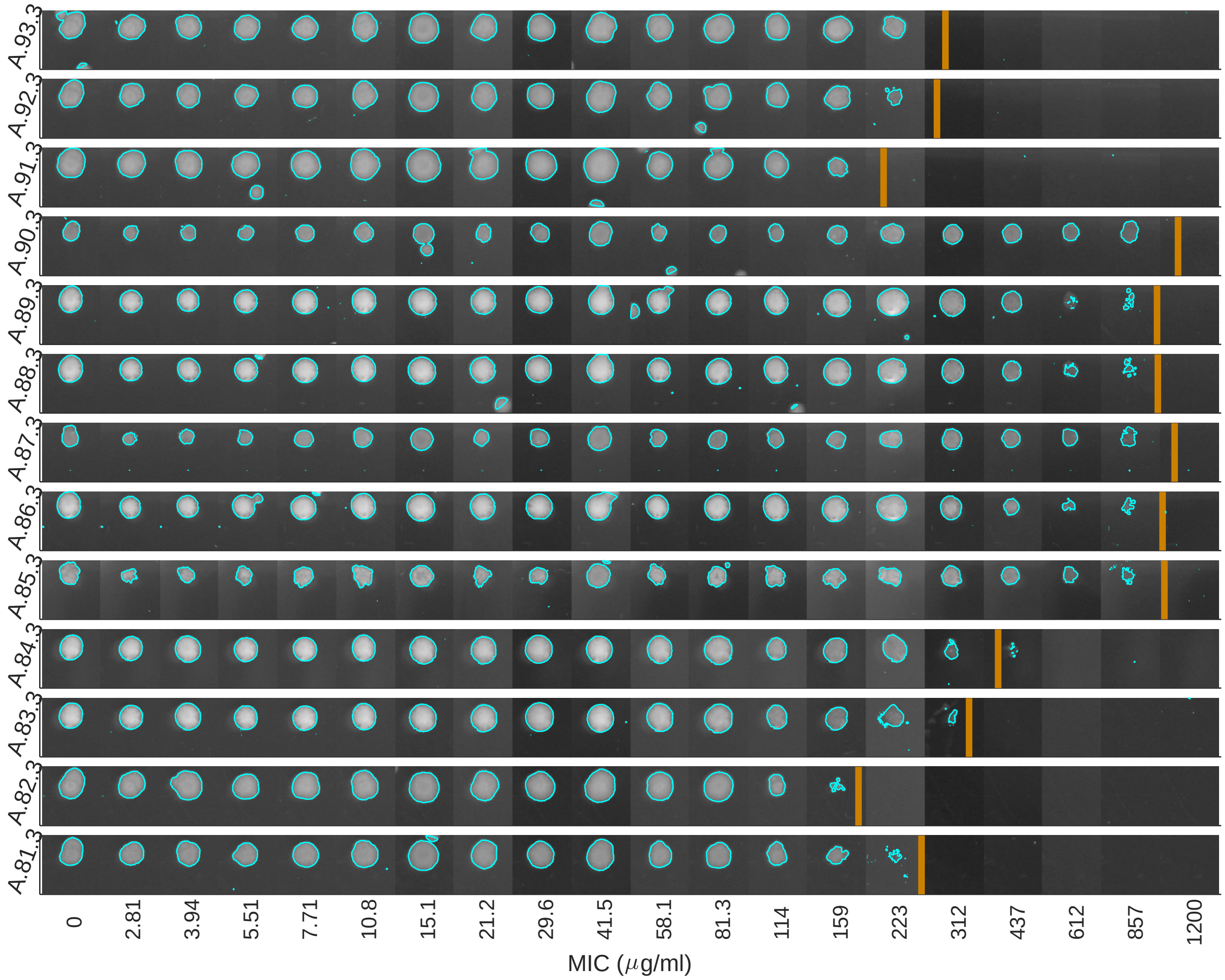

Supplementary Datasets 1: Ampicillin resistance measurements for isolates collected in Experiment A.
